# The induction of a mesenchymal phenotype by platelet cloaking of cancer cells is a universal phenomenon

**DOI:** 10.1016/j.tranon.2021.101229

**Published:** 2021-09-27

**Authors:** Cathy D. Spillane, Niamh M. Cooke, Mark P. Ward, Dermot Kenny, Gordon Blackshields, Tanya Kelly, Mark Bates, Yanmei Huang, Cara Martin, Sinead Skehan, Aoife Canney, Michael Gallagher, Paul Smyth, Nathan Brady, Andres Clarke, Bashir Mohamed, Lucy Norris, Doug A. Brooks, Robert D. Brooks, Jessica K. Heatlie, Stavros Selemidis, Sean Hanniffy, Eric Dixon, Orla Sheils, Sharon A. O'Toole, John J. O'Leary

**Affiliations:** a Department of Histopathology, Trinity College Dublin and Trinity St James's Cancer Institute, Dublin, Ireland; b Emer Casey Molecular Pathology Research Laboratory, Coombe Women and Infants University Hospital, Dublin, Ireland; c The Biomedical Diagnostics Institute, Dublin City University, Dublin, Ireland; d Department of Molecular and Cellular Therapeutics, Royal College of Surgeons in Ireland, 123 St Stephens Green, Dublin, Ireland; e School of Forensic Medicine, Xinxiang Medical University, Xinxiang, Henan, China; f Department of Obstetrics and Gynaecology, Trinity College Dublin, Trinity Centre for Health Sciences, St James's Hospital, Dublin, Ireland; g Cancer Research Institute, University of South Australia, Adelaide 5001, Australia; h School of Health and Biomedical Sciences, RMIT University, Bundoora, Victoria 3083, Australia; i BD Research Centre Ireland, Limerick, Ireland; j BD Technologies and Innovation, Research Triangle Park, NC, USA

**Keywords:** Cancer, Platelets, Metastasis, 5-gene panel, PAI-1

## Abstract

Tumour metastasis accounts for over 90% of cancer related deaths. The platelet is a key blood component, which facilitates efficient metastasis. This study aimed to understand the molecular mechanisms involved in tumour-platelet cell interactions.

The interaction between cancer cells and platelets was examined in 15 epithelial cell lines, representing 7 cancer types. Gene expression analysis of EMT-associated and cancer stemness genes was performed by RT-PCR. Whole transcriptome analysis (WTA) was performed using Affymetrix 2.0ST arrays on a platelet co-cultured ovarian model.

Platelet adhesion and activation occurred across all tumour types. WTA identified increases in cellular movement, migration, invasion, adhesion, development, differentiation and inflammation genes and decreases in processes associated with cell death and survival following platelet interaction. Increased invasive capacity was also observed in a subset of cell lines. A cross-comparison with a platelet co-cultured mouse model identified 5 common altered genes; PAI-1, PLEK2, CD73, TNC, and SDPR.

Platelet cancer cell interactions are a key factor in driving the pro-metastatic phenotype and appear to be mediated by 5 key genes which have established roles in metastasis. Targeting these metastasis mediators could improve cancer patient outcomes.

## Highlights

• Platelet cancer cell interactions are a key factor in driving the pro-metastatic phenotype.
• Platelet cancer cell interactions appear to be mediated by 5 key genes which have established roles in metastasis.
• Targeting these mediators of metastasis could improve outcomes for cancer patients.

## Introduction

Tumour metastasis is the pivotal contributory factor in determining prognosis for cancer patients, accounting for over 90% of all cancer related deaths [1]. Cancer metastasis involves a cell or group of cells exiting the primary tumour via the induction of “epithelial mesenchymal transition” (EMT), and intravasating into the vasculature where they are known as circulating tumour cells (CTCs) [2,3]. These aggressive cancer cells must protect themselves in the vascular system during this trafficking phase; to avoid immune detection and shear stress and to arrive viable and metabolically active at a secondary site where extravasation and the establishment of a secondary metastatic tumour can occur [4].

The platelet is a key blood component involved in facilitating efficient metastasis, which may involve a physical cloak or other immunomodulatory functions. Cancer animal model studies have shown that platelet depletion, anti-GPIIb/IIIa platelet receptor blockade [5] and Nbeal2-/- [gray platelet syndrome [GPS] murine model] significantly reduce metastatic disease [6]. Consequently, *ex-vivo* pre-treatment of platelets with aspirin and P2Y12 inhibitors, significantly reduces the formation of tumour cell-induced platelet aggregates (TCIPAs) and thereby reduces cancer cell invasion [7,8]. Epidemiology studies have shown that aspirin reduces cancer incidence and mortality and specifically that metastatic disease is minimised in patients who were taking aspirin prior to diagnosis [9,10]. Our group has previously shown that cancer patients have high platelet counts, and that their platelets are often highly prone to activation and auto-aggregation [11]. Furthermore, endogenous CTC clusters/micro-emboli composed of blood components including platelets have been shown to have increased metastatic potential compared to CTC singlets [2] and to greatly increase the risk of patient death.

Platelets are necessary components of the secondary metastatic niche [4] and platelet-cancer cell interactions aid survival of cancer cells *via* the inhibition of natural killer cell activity [12], whilst providing protection from shear stress in the circulation [13]. Our recent review outlines in detail the molecular role that platelets, immune cells and the coagulation cascade play in CTC biology, impacting on EMT, pro-survival signalling and immune evasion [14]. Consequently, we believe that platelet cloaked CTCs have a higher propensity towards metastasis, and the protection conferred by platelets also assists their extravasation at the secondary metastatic site. Here we demonstrate that activated platelets have a high affinity for cancer cells, regardless of the cancer type; and that they induce and maintain EMT which can protect these tumour cells from environmental stress while simultaneously assisting in immune evasion. Understanding the molecular mechanisms involved in tumour-platelet cell interactions and where this interaction is initiated is key to the elimination of CTCs and the prevention of metastasis.

## Materials and methods

*Ethics statement:* Blood collection for this study was approved by the Royal College of Surgeons in Ireland and the Coombe Women & Infants University Hospital ethics committee. Healthy donors who had not taken medications known to affect platelet function for ≥10 days were recruited. Written informed consent was obtained from all donors prior to phlebotomy. Subjects were not assigned to experimental groups using simple randomisation because this was not applicable to this study. Each participant was given a unique identification number, blinding was not applicable. As experiments were cell line based, no power analysis was performed. Sex as a biological variable was considered but based on 59 M cell data, no difference was observed between male and female donor.

*Cell lines:* Fifteen cancer cell lines of epithelial origin were included in this study. All cell lines were maintained in appropriate culture media as recommended by source supplier (Supplementary Table 1), supplemented with 10% fetal bovine serum (FBS), 2 mM L-glutamine and 2% penicillin/streptomycin unless stated otherwise in Supplementary Table 1. All cell lines were grown in standard conditions and maintained in a cell culture incubator at 37 °C containing 5% CO_2_. From thawing to maximum use was within 10–12 passages, biological replicate experiments were within 5 passages. Routine mycoplasma testing is performed in the laboratory every 3 to 6 months using the LookOut Mycoplamsa PCR kit (Sigma-Aldrich, United Kingdom). Images of cells were taken using an axiovert 35 inverted phase contrast microscope (Zeiss, Germany) and a Canon EOS 700D camera.

*Platelet Preparation:* Whole blood was collected by venipuncture through a 19-gauge butterfly needle without a tourniquet to avoid platelet activation. Platelet-rich plasma (PRP) was prepared from 3.2% trisodium citrated blood (10% vol/vol) centrifuged at 170 g for 10 min. For the preparation of washed platelets, blood was collected into Acid-Citrate-Dextrose (ACD: 38 mM citric acid, 75 mM sodium citrate, 124 mM D-glucose) which acted as an anticoagulant (15% vol/vol) and centrifuged at 170 g for 10 min. PRP was acidified to pH 6.5 with ACD, and PGE1 (1 µM) was added to avoid platelet activation during centrifugation. Platelets were pelleted by centrifugation at 720 g for 10 min. The supernatant was removed, and the platelet pellet was resuspended in JNL buffer [130 mM NaCl, 10 mM sodium citrate, 9 mM NaHCO_3_, 6 mM D-glucose, and 0.9 mM MgCl_2_, 0.81 mM KH_2_PO_4_, and 10 mM Tris, pH 7.4] and supplemented with 1.8 mM CaCl_2_. All platelets’ samples were processed within 60 min of venipuncture.

*Platelet adhesion assay:* Platelet adhesion to cancer cells was measured by flow cytometry. The assay was based on the detection of a platelet specific marker CD42b, (GPIbα) (Clone HIP1) (BD Biosciences Cat# 551061, RRID: AB_398486) on the surface of cancer cells following co-incubation. Washed suspensions of cancer cells (1 × 10^6^/mL) were incubated with PRP from a single donor resulting in a cancer cell:platelet ratio of 1:1000 for 1 min under low shear conditions (rocking table, 12 o.p.m.). At this ratio, no tumour cell-induced platelet aggregation is observed, but there is efficient coating of tumour cells by platelets with a degranulated phenotype [15]. Next, samples were washed to remove surplus platelets and soluble factors, fixed with 3.7% paraformaldehyde, blocked with 1% BSA and labelled with either an allophycocyanin (APC)-labelled mouse anti-human CD42b antibody (BD Biosciences Cat# 551061, RRID: AB_398486) or isotype control (BD Biosciences). Samples were analysed within 1 h by flow cytometry using a BD FACS Canto and data was analysed using BD FACS DIVA^TM^ software (Becton Dickinson, Palo Alto, CA, USA). Gating strategy is displayed in Supplementary Fig. 1.

*Platelet activation assay:* Platelet activation by cancer cells was measured by a flow cytometry assay, based on the detection of P-selectin; CD62P (BD Biosciences Cat# 550888, RRID: AB_398475) on the surface of platelets following co-incubation. P-selectin is stored internally in alpha-granules of resting platelets and is translocated to the surface upon activation. Washed suspensions of cancer cells (1 × 10^6^/mL) were incubated with PRP resulting in a cancer cell:platelet ratio of 1:30 for 15 min under low shear conditions (rocking table, 12 o.p.m.). The reaction was terminated with 1 mL of JNL buffer. Samples were processed as described above and labelled with either an APC-labelled mouse anti-human P-selectin antibody (BD Biosciences Cat# 550888, RRID: AB_398475) or isotype control (BD Biosciences). Gating strategy is displayed in Supplementary Fig. 2.

*Real Time PCR:* Total RNA was extracted from the samples using the miRVana Kit (Life Technologies, Foster City, CA, USA), according to the manufacturer's protocol. The mRNA expression level of genes of interest was profiled using TaqMan RT-PCR technology, either using standard assays or a custom designed OpenArray® Panel (Life Technologies, Foster City, CA, USA). For the standard assays, cDNA template was prepared using the high capacity cDNA archive kit (Life Technologies, Foster City, CA, USA). When using the OpenArray® Panels, cDNA template was prepared from 2.5 μg of total RNA using the high capacity cDNA archive kit. The samples were next subjected to real time-PCR analysis using TaqMan mRNA primers and probes on the TaqMan OpenArray platform. Ct (crossing threshold) values for each mRNA target were calculated using the OpenArray® Real-Time qPCR analysis software. For all assays, expression values were calculated using the comparative Ct method, with GAPDH or B2M used as the endogenous control.

*Cancer Cell Invasion Assay:* BioCoat® Matrigel® (Corning) invasion chambers (8 μm pore size) were used to examine the effect of platelets on cancer cell invasion. MCF-7, PC-3 and SiHa cancer cells were incubated with media alone or washed platelets (final cancer cell-platelet ratio 1:1000) for 24 h. After incubation, platelets were removed by washing cells in PBS. Cell suspensions were prepared in serum-free medium (5 × 10^4^ cells/ml) and allowed to invade through Matrigel® for 24 h at 37 °C in 5 % CO_2_. Bottom wells contained 10 % FBS as a chemoattractant. Invaded cells were fixed (methanol) and stained with crystal violet. Filters were imaged using a Cell Imaging System (EVOS® FL) for subsequent analysis and quantified with Image J (NIH). The number of cells per 4 × high power field (cells/hpf) was determined in 3 random hpf, and the mean number of cells/hpf was calculated for each membrane. All experiments were carried out with a minimum of three wells per condition.

*Whole transcriptome array analysis:* For these experiments total RNA was isolated from washed platelet-treated or untreated SKOV3 or 59M cells (*n* = 3). The concentration of RNA isolated from platelets alone was below detection limits and could therefore not be used for microarray analysis. In order to detect mRNAs contributed by platelets, RNA was also isolated from cells incubated with washed platelets for ≤1 min in suspension (*n* = 3). 100 ng of total RNA from each sample underwent cDNA synthesis, followed by a cleanup, overnight IVT amplification, a second round of cDNA synthesis and a cleanup using the Ambion® WT Expression Kit (Life Technologies, Foster City, CA, USA), according to manufacturer's instructions. The cDNA was then fragmented and labelled using the Affymetrix® GeneChip® WT Terminal Labeling Kit, according to manufacturer's instructions. Single stranded fragmented, biotin labelled DNA was hybridised for 17 h to GeneChip® Human Gene 2.0 ST arrays (Affymetrix®, Santa Clara, CA, USA). Hybridised arrays were scanned on an Affymetrix GeneChip® Scanner 3000 7G (Affymetrix®, Santa Clara, CA, USA). The Affymetrix microarray data sets generated as part of this study are available in the ArrayExpress (ArrayExpress, RRID:SCR_002964) repository, accession #452909.

*Bioinformatics:* Arrays were examined using quality control methods as outlined in the quality assessment white paper (Affymetrix, 2007). Gene array data was analysed using Bioconductor (Bioconductor, RRID:SCR_006442) software libraries [16] and the RMA method [17]. Differential expression analysis across all the arrays was carried out using RankProd (RankProd, RRID:SCR_013046) [18]. Differentially expressed data was further interrogated using DAVID (DAVID, RRID:SCR_001881) analysis to identify molecular function and biological process-related genes through gene ontology [19], and IPA (QIAGEN Inc., https://www.qiagenbioinformatics.com/products/ingenuitypathway-analysis) to identify upstream regulator and regulatory pathways.

*Epidermal Growth Factor (EGF) Treatment:* On day 0 for each replicate of the EMT/MET model, cells were split (SKOV3, 1:8 and 59 M, 1:15) into six T75 flasks; 2 flasks remaining untreated in normal culture media (NCM), 2 flasks were treated with 10 ng/mL EGF (Epidermal Growth Factor human, recombinant expressed in E. coli (Sigma E9644)) and 2 flasks treated with the vehicle control, Acetic Acid (AA). The cells received this treatment for 6 days. On the 6th day of treatment, one flask from each experimental condition was pelleted for genes expression analysis. The remaining flasks were then passaged using the same split ratios as above for each cell line. For cells grown in NCM they continued to be grown in NCM. Cells grown in NCM supplemented with EGF were allocated to a flask containing either NCM supplemented with 10 ng/mL EGF or a 2nd flask containing only NCM (EGF/M). The AA treated cells were treated in a similar manner with cells allocated between a flask containing NCM supplemented with AA and one containing only NCM (AA/M). After a further 6 days of culture the cells were pelleted for gene expression analysis.

*Wound Healing Assay:* SKOV3 and 59M were seeded in 6-well plate at a concentration of 7.5 × 10^4^ cells/well and 2.0 × 10^4^ cells/well, respectively. On day 5, media containing 10% FBS was removed and the cells were washed with PBS. The appropriate media for each well was added containing 0% FBS and left overnight. On day 6, a wound was scratched into the cell monolayer using a sterile tip. The cells were left settle for 1 h before taking images of the scratched area, time point 0. Further images were taken at 6, 24 and 48 h time intervals. Images were analysed and the area of each scratch calculated using the MRI Wound Healing Tool add-on in ImageJ (ImageJ, RRID:SCR_003070) software.

*Statistics:* Statistical significance was determined using an unpaired student's *t*-test. A statistically significant difference was considered to be present when *p* ≤ 0.05. Statistical analyses were performed using either Microsoft excel (Microsoft Excel, RRID:SCR_016137) or GraphPad Prism 7 software (GraphPad Prism, RRID:SCR_002798).

## Results

### Universal platelet adhesion to epithelial cancer cells

**Fig.1:**
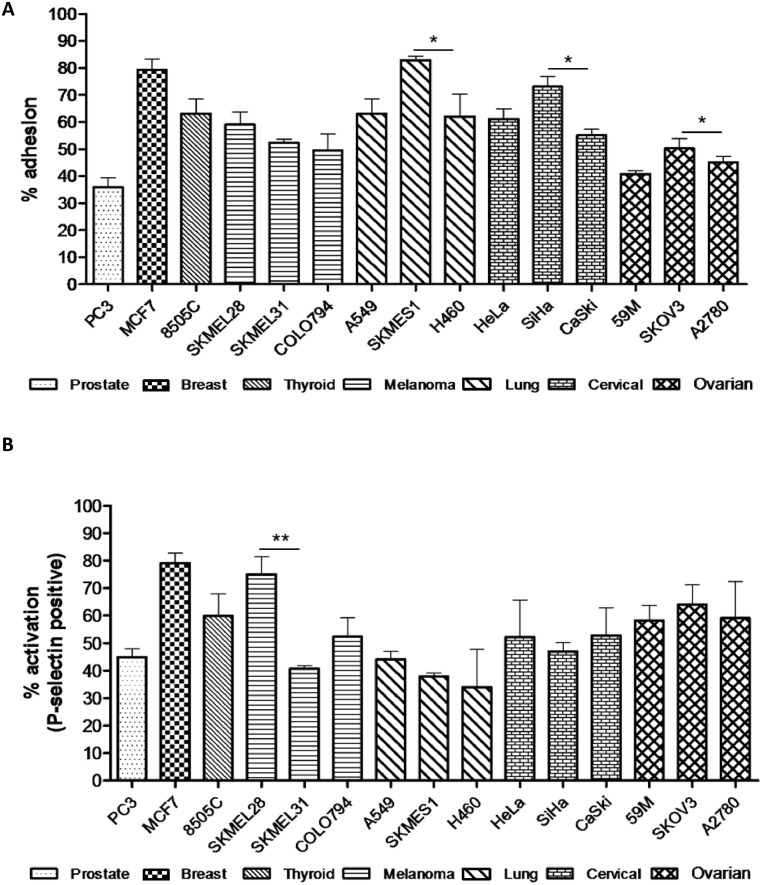
**Platelet interactions with cancer cells are universal but heterogeneous. (A)** Platelet adhesion to cancer cells was quantified based on the fluorescence detection of CD42b positive platelets (*n* = 4). **(B)** Platelet activation by cancer cells was quantified based on the fluorescence detection of P-selectin (CD62P) positive platelets (*n* = 3). Significant variations were seen between cell some lines from the same cancer type. Data shown are mean values + SD. * *p ≤* 0.05, ** *p ≤* 0.01 was determined by Student's *t* test, with significant differences between cell lines of the same tumour type highlighted.

Previously, we described the interaction of platelets with ovarian cancer cells [7,20]. To further our understanding of the importance of this interaction, we determined whether this phenomenon was observed with other epithelial cell derived cancers. The adhesion rate between platelets and 15 human cancer cell lines of different cancer origin and metastatic potential was examined using flow cytometry (Fig. 1A). This involved the detection of the platelet marker CD42b on cancer cells post-incubation with platelets and subsequent washing. Platelet adhesion or ‘cloaking’ of these epithelial cancer cells occurred across all of the 7 tumour types examined; breast, cervix, lung, melanoma, ovary, prostate and thyroid. However, the level of platelet adhesion varied, ranging from 35% in PC-3 cells (metastatic prostate cancer) to 83% in SK-MES-1 cells (metastatic lung squamous cell carcinoma). With the exception of melanoma, significant variation was also seen within cell lines of the same cancer type (Fig. 1A). Interestingly, these variations did not appear to correlate with the primary or metastatic site origin of the cell lines, or with whether the cancer type was originally considered to have high or low CTC trafficking potential [21,22]. There was, however, a general trend for cell lines within a cancer type to behave similarly relative to other cancer types. For example, cell lines from cervix and lung exhibited similarly higher adhesion rates when compared to those from ovary (Fig. 1A). Next, we examined if this interaction resulted in the activation of the platelets, measured via detection of P-selectin on the platelet surface using flow cytometry. All 15 cell lines induced platelet activation with levels varying both across and within tumour types (Fig. 1B). There was no apparent correlation between the level of platelet adhesion and activation (Fig. 1 and Supplementary Fig. S3). While these results indicate that the degree of physical interaction between platelets and cancer cells and the subsequent activation of the platelets is heterogeneous, they do suggest that aggressive cancer cell platelet interaction is a universal phenomenon.

### Platelets induce a mesenchymal-like phenotype in epithelial cancer cells

**Fig.2:**
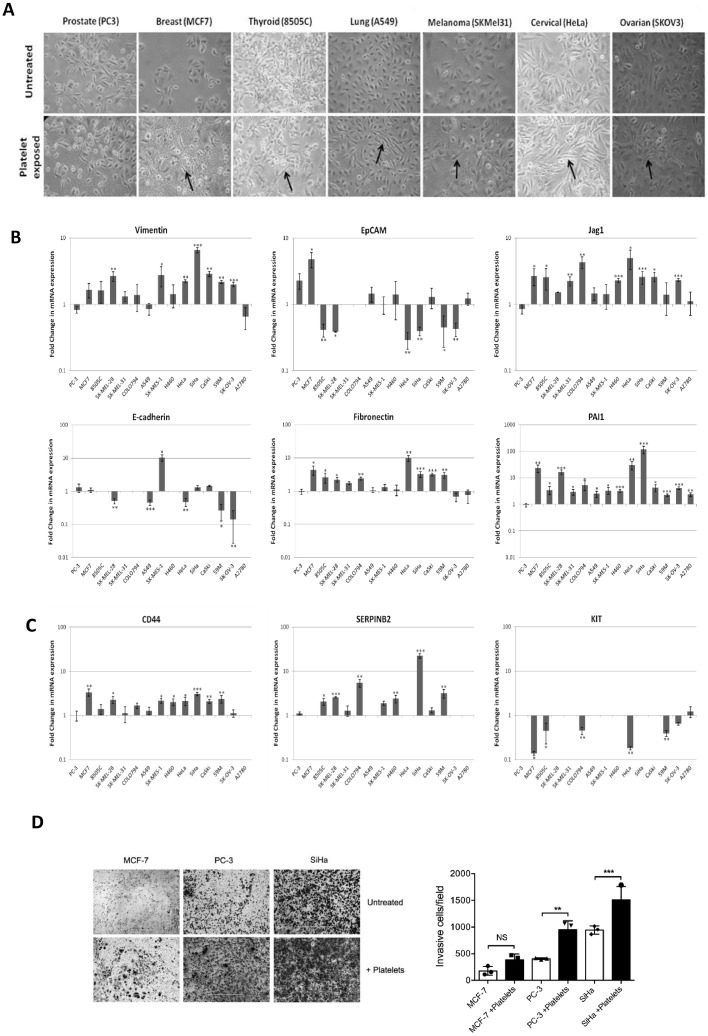
**Platelets induce a mesenchymal-like state in cancer cells.** (A) Phase-contrast micrographs of cancer cells grown in the absence or presence of platelets for 24 h. (B) Gene expression profile of EMT related genes in platelet cloaked cancer cells (note scale differences representing more abundantly expressed genes). (C) Gene expression profile of cancer stemness related genes in platelet cloaked cancer cells. Results are expressed as fold change in mRNA expression in cancer cells in the presence of platelets relative to control cells grown in the absence of platelets for 24 h (*n* = 3). (D) Effect of platelets on cancer cell (MCF-7, PC-3 and SiHa) invasion. Data are expressed (*n* = 3) as number of cells invaded in field through Matrigel® chambers relative to the control. Values are normalised to endogenous control. Values are normalised to endogenous control. Data shown are mean value + SD. *** *p* ≤ 0.01, ** *p* ≤ 0.01, * *p* ≤ 0.05 was determined by Student's *t* test.

We next sought to understand whether the observed cancer cell interaction extended beyond platelet adhesion and activation to the induction of common molecular mechanisms within the cancer cells. Previously, we demonstrated the ability of platelet adhesion to induce an EMT signature in ovarian cancer cells [7,20]. To assess if this was also universal, all 15 cancer cell lines were co-cultured with platelets for 24 h and their subsequent morphological and gene expression profiles analysed. Morphological alterations, including loss of cell-cell contact and elongation of shape, indicative of a more mesenchymal-like phenotype were observed in all of the cancer cell lines compared to their untreated counterparts; with the exception of the PC-3 prostate cancer cells, which interestingly already tend to adopt this morphological phenotype in culture (Fig. 2A).

Gene expression analysis of a panel of 9 well characterised EMT-associated genes and 4 genes known to have a dual role in EMT and cancer stemness was performed by RT-PCR (significant differences were defined as fold change ≥ ±2 and *p-*value ≤ 0.05). There were significant increases in the expression of the mesenchymal markers vimentin (VIM) and fibronectin 1 (FN1), and the EMT associated signalling protein, jagged 1 (JAG1) in over 50% of the cell lines (Fig. 2B). Additionally, decreases in the expression of the epithelial markers E-cadherin (CDH1) and epithelial cell adhesion molecule (EpCAM) were observed in approximately 50% of the cell lines in which there was endogenous expression of these genes prior to platelet co-culture (Fig. 2B). While there was no consistent pattern to the EMT associated changes, >85% (13/15) of cell lines examined had 2 or more gene expression alterations indicative of an increased mesenchymal phenotype. The expression of the 4 cancer stem cell associated genes was also modulated by the interaction, with an increase in the expression of the cancer stem cell associated markers CD44 and SERPINEB2, in 8 and 6 of the 15 cell lines respectively, and a decrease in KIT in 5 of the 15 cell lines respectively (Fig. 2C). The effects of platelets on the epithelial phenotype of the cancer cells again highlights the universal yet heterogeneous nature of this interaction, with varying degrees of influence occurring across different cancer types.

To build on our previous findings of platelets increasing invasion in ovarian cancer (7) and to assess if the genotypic changes observed in mesenchymal markers resulted in increased metastatic potential, we interrogated a subset of cancer cell lines (breast; MCF-7, prostate; PC-3 and cervical; SiHa) for invasive capacity following platelet exposure. Interestingly, exposure to platelets increased PC-3 and SiHa cell invasion (Fig 2D, *p* = 0.0003; *p* > 0.0001). A trend was seen towards increased invasion in the MCF-7 cell line; however, this did not reach statistically significance (*p* = 0.1567).

### Platelets maintain an EMT transition in cancer cells

It is generally accepted that CTCs undergo EMT in order to leave the tumour environment and enter into the circulation and that they require this phenotype to some degree in order to establish a tumour in a secondary environment. To investigate whether platelets could facilitate the maintenance of this more motile phenotype, we developed an EMT/mesenchymal epithelial transition (MET) model in two of the ovarian cancer cell lines (59M and SKOV3), using epidermal growth factor (EGF) treatment, which was previously shown to upregulate EMT markers, inhibit E-cadherin expression, and promote EMT and cancer cell migration/invasion. The rationale for selecting these cell lines was derived from previous results based on the expression of epithelial and mesenchymal markers, as well as their invasive capacities, which demonstrated 59M cells to exhibit a more mesenchymal-like profile than SKOV3 cells [7].

**Fig. 3:**
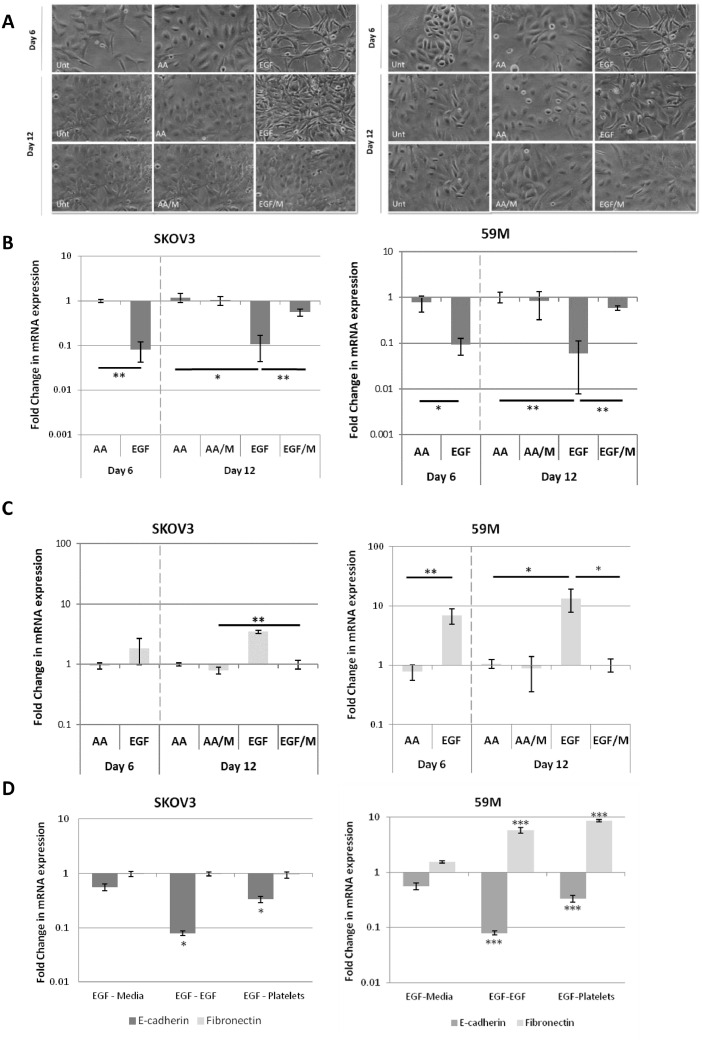
**The effect of EGF treatment on the expression of the epithelial and mesenchymal markers in SKOV3 cells.** SKOV3 and 59M cells were treated for 6 days with either 10 ng/mL EGF, AA or left untreated. On day 6 cells grown in EGF were continued in media with EGF (EGF) or without EGF (EGF/M). Cells grown in AA were continued to be grown in media with AA (AA) or without (AA/M). **(A)** Phase-contrast micrographs at 6 and 12 days after treatment. **(B)** SKOV3 and 59M cells; results are expressed as fold changes in mRNA expression of E-cadherin in the presence of EGF or AA (vehicle control) compared to untreated cells (*n* = 3). Values were normalised to the endogenous control B2M. **(C)** SKOV3 and 59M cells; results are expressed as fold changes in mRNA expression of Fibronectin in the presence of EGF or AA (vehicle control) compared to untreated cells (*n* = 3). Values were normalised to the endogenous control B2M. **(D)** SKOV3 and 59M EGF treated cells subsequently cultured in normal culture media with or without EGF or platelets; results are expressed as fold changes in mRNA expression. Values were normalised to the endogenous control B2M. Data shown are mean values ± SD (*n* = 3). Significance was determined using Student's *t* test, where *p ≤ 0.05. ** *p* ≤ 0.01, *** *p* ≤ 0.001.

The model was validated by morphology, PCR and cell migration analysis (Figs. 3 and S4–7). The results demonstrated that the induction of EMT could be achieved in ovarian cancer cell lines through treatment with EGF and that returning the cells to normal culture media enabled MET to occur. By treating SKOV3 and 59M cells with EGF over a 6-day period, we noted a dramatic change in morphology compared to controls (untreated and acetic acid treated cancer cells), with cells losing their rounded, cobblestone appearance to become elongated with marginal cell-cell contacts (Fig. 3A).

EGF treatment resulted in a significant decrease in E-cadherin expression in both SKOV3 and 59M cells (Fig. 3B). By comparison, while EGF-treatment did not significantly alter fibronectin expression in SKOV3 cells, its expression was significantly increased (*p* < 0.001) in EGF-treated 59M cells (Fig. 3C). EGF promotion of EMT was also evident in the trend towards increased expression of the mesenchymal markers Vimentin in the SKOV3 cells and Claudin in the 59M cells (Supplementary Figs. S4 and S5). Importantly, this EMT signature was no longer evident upon further culturing of these cells in the absence of EGF (Fig. 3B,C and Supplementary Figs. S4 and S5) suggesting an ability to revert to an epithelial phenotype. Wound healing assays also showed that SKOV3 cells exhibit increased motility after EGF treatment (Supplementary Fig. S6). The same could not be demonstrated for the 59M cell line, as this cell line is an *ab-initio* metastatic highly aggressive cell line [7] (Supplementary Fig. S7).

The model was then employed to investigate the ability of platelets to maintain the EGF-induced mesenchymal-like phenotype. Gene expression analysis demonstrated that EGF-treated cells subsequently co-cultured with platelets had a similar expression profile of fibronectin and E-cadherin to cells maintained in EGF supplemented media (Fig. 3D). While cells previously treated with EGF and then grown in the absence of EGF exhibited an expression profile suggestive of MET, with no significant difference observed in the gene expression of fibronectin and E-cadherin compared to cells maintained in normal culture media. These experiments demonstrate that cells grown in the continued presence of EGF or co-cultured in the presence of platelets were able to maintain their EMT signature and associated mesenchymal-like phenotype, while cells grown in the absence of either reverted to an epithelial-like phenotype.

### The interaction between cancer cells and platelets drives a pro-metastatic microenvironment

To gain a broader picture of the molecular signalling pathways involved in the communication between epithelial cancer cells and platelets, we determined the platelet induced gene expression signature in epithelial cancer cells using whole transcriptome microarray analysis. This analysis was performed on two ovarian cancer cell lines, 59M and SKOV3 due to their inherent properties outlined above. By evaluating these two cells lines, we aimed to identify the key genes being altered in cancer cells following their interactions with platelets. Therefore, examining the overlapping genes from each cell type might enable the identification of a more universal gene signature of the EMT process and/or mesenchymal state.

Transcriptome expression profiles of cancer cells co-cultured with platelets were compared to those left untreated over the same time period; and significant differences in gene expression were defined as fold change ≥ ±1.8 and FDR ≤ 0.05. To exclude the possibility that platelet mRNA may contribute to the gene signatures produced, microarray analysis was performed to identify platelet specific mRNAs. In total 6 platelet mRNAs contributed a significant level to the produced gene signatures (TGM2, AMTN, PKIA, PPBP, LHX1 and CTD-2287016.3) and were excluded from subsequent analyses.

**Fig.4:**
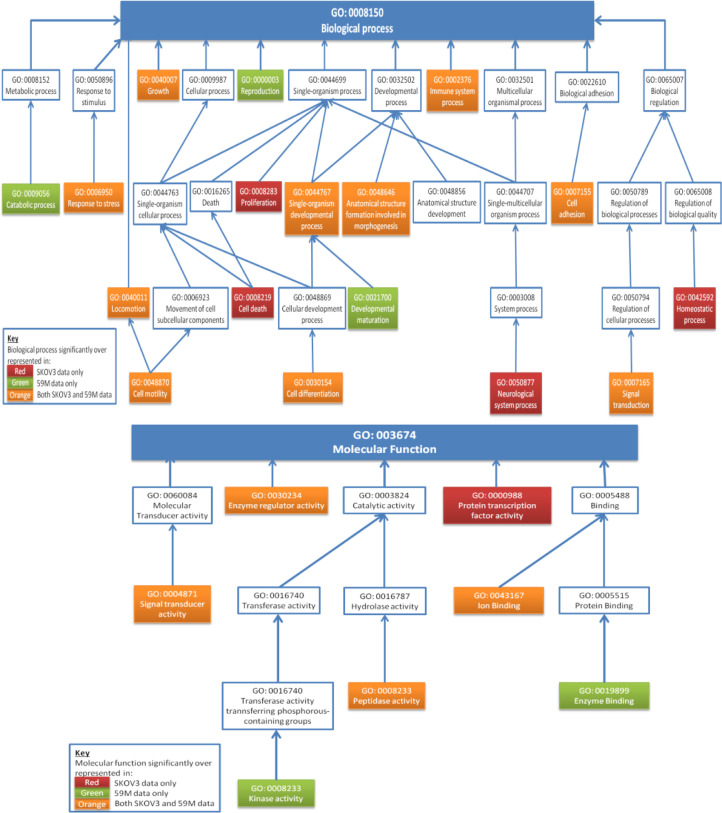
**Platelets induce similar phenotype changes in different cells lines.** Gene Ontology **(A)** biological processes and **(B)** molecular functions significantly associated with the list of genes differentially expressed in SKOV3 (red), 59M (green) or both cell lines (orange) upon platelet treatment (platelet induced gene signature; threshold = 1.8-fold, up and down-regulated genes considered, FDR ≤ 0.05) as determined with DAVID Bioinformatics Resource. (For interpretation of the references to colour in this figure legend, the reader is referred to the web version of this article).

The interaction between platelets and SKOV3 cells appeared to have a more pronounced effect, with 129 genes significantly altered compared to 80 in the 59M cells (Table S2). However, gene ontology analysis demonstrated that the molecular mechanism induced by these gene alterations were similar for both cell lines, with a high correlation between the biological processes, molecular functions and pathways affected (Fig. 4, Table 2). This included an increase in processes associated with cellular movement, migration, invasion, adhesion, development, differentiation and inflammation and a decrease in processes associated with cell death and survival. A cross-comparison of the data sets identified 34 genes commonly and significantly altered in both cell lines (Table 1). Analysis of this smaller gene set demonstrated a similar gene ontology profile to the analysis of individual gene sets, therefore further validating the importance of this gene set in translation of the effects induced by the platelets. Identification of the upstream regulators of each data set identified again an overlapping pattern between the SKOV3 and 59M gene signatures and that this pattern was maintained when the panel of 34 common genes was analysed (Table 2B). From the upstream regulator analysis, it was possible to identify key regulators, such as TGFβ1, TNF, HIF1A, EGF and EDN1, all of which are known to play a prominent role in EMT, extracellular matrix (ECM) remodelling and inflammation. Examples of some of the key regulators such as TGFβ1 and TNF are displayed in Supplementary Fig. S8. Thus, the bioinformatics analysis demonstrated that the biological processes induced by platelet adhesion to the cancer cells were potentially contributing to the formation of a pro-metastatic environment.

**Table 1:** Gene list of 34 common differentially expressed genes.

Expression in:		SKOV3		59M	
Term	Entrez gene ID	Fold Change	FDR	Fold Change	FDR
LAMC2	3918	7.8	4.4E-12	5.8	7.21E-05
MMP2	4313	2.4	6.6E-05	3.0	1.29E-04
PMEPA1	56937	2.7	7.5E-06	3.0	2.72E-04
NT5E	4907	2.3	4.7E-05	2.9	3.02E-04
RP11-597K23.2	1.02E+08	3.0	2.0E-05	2.9	3.02E-04
TFPI2	7980	2.8	1.5E-06	2.8	6.94E-04
MMP1	4312	1.9	3.6E-03	2.7	4.67E-03
DOCK4	9732	1.8	8.7E-03	2.6	2.72E-04
SEMA7A	8482	2.4	1.8E-04	2.5	2.07E-03
G0S2	50486	2.3	6.5E-05	2.3	8.29E-03
NRP2	8828	2.0	8.2E-04	2.3	7.73E-03
PLAUR	5329	2.7	2.1E-06	2.3	6.94E-04
TGFBI	7045	2.2	2.4E-04	2.2	6.94E-04
ADAM19	8728	4.7	1.9E-09	2.2	2.60E-03
DNER	92737	2.2	3.5E-04	2.2	1.43E-03
SERPINE1	5054	2.7	6.1E-06	2.2	7.27E-04
EPHB2	2048	2.2	8.6E-05	2.1	6.94E-04
LAMB3	3914	3.2	4.6E-07	2.0	1.56E-02
HIC1	3090	1.8	1.9E-02	1.9	1.49E-02
TNC	3371	3.1	9.2E-07	1.9	1.88E-03
PLEK2	26499	2.6	8.3E-06	1.9	2.26E-02
PLAU	5328	2.3	5.9E-05	1.8	1.60E-02
ANPEP	290	1.9	3.3E-03	1.8	5.94E-03
SEMA3C	10512	1.8	3.9E-03	1.8	2.04E-02
PTGIS	5740	-3.2	5.3E-07	-1.8	1.67E-02
EGR1	1958	-1.9	7.6E-03	-1.8	8.29E-03
ID1	3397	-2.3	3.5E-04	-1.9	5.39E-03
SDPR	8436	-2.2	1.4E-04	-1.9	3.46E-03
CITED2	10370	-3.2	4.6E-07	-1.9	6.35E-03
PEG10	23089	-2.5	1.4E-04	-2.0	6.94E-04
NQO1	1728	-1.8	6.1E-03	-2.1	1.06E-02
CPA4	51200	-1.8	4.3E-03	-2.3	6.94E-04
ITGB8	3696	-2.3	6.9E-05	-3.0	3.04E-04
CYP1B1	1545	-3.5	2.6E-07	-3.2	2.72E-04

**Table 2A:** Enriched KEGG pathways.

SKOV3 data set (129 genes)		59M data set (80 genes)	
Term	*p*-value	Term	*p*-value
ECM-receptor interaction	2.40E-08	Bladder cancer	1.60E-04
Small cell lung cancer	3.90E-06	ECM-receptor interaction	2.10E-04
Pathways in cancer	1.20E-05	Focal adhesion	1.90E-03
Focal adhesion	1.20E-05	TGF-beta signalling pathway	2.50E-03
Hypertrophic cardiomyopathy (HCM)	3.70E-04	Pathways in cancer	5.20E-03
Arrhythmogenic right ventricular cardiomyopathy	1.60E-03	Small cell lung cancer	1.80E-02
Hematopoietic cell lineage	2.80E-03	Hematopoietic cell lineage	1.90E-02
Dilated cardiomyopathy	3.70E-03	Cytokine-cytokine receptor interaction	2.90E-02
Complement and coagulation cascades	7.60E-03	p53 signalling pathway	7.50E-02
Toll-like receptor signalling pathway	2.70E-02

**Table 2B:** Top 10 upstream regulators (Activation z-score >2 and *p*-value <0.05).

SKOV3 data set (129 genes)			59M data set (80 genes)			Overlapping data set (34 genes)		
Upstream regulator	Activation *z*-score	*p*-value	Upstream regulator	Activation *z*-score	*p*-value	Upstream regulator	Activation *z*-score	*p*-value
TGFB1	4.709	3.66E-37	TGFβ1	4.127	3.78E-22	TGFB1	4.139	3.12E-27
TNF	3.208	8.83E-32	TNF	3.863	6.44E-19	TNF	4.028	2.80E-21
ERBB2	2.199	5.47E-22	RGF	2.89	1.47E-15	ERBB2	2.802	2.86E-20
EDN1	2.686	1.80E-17	Ras	2.391	2.03E-14	Cg	2.576	5.58E-17
F2	2.648	7.66E-17	EDN1	2.453	2.60E-14	EDN1	2.795	2.57E-16
ER	-3.071	9.22E-17	FGF2	2.866	1.15E-13	EGF	2.074	6.04E-15
Cg	2.763	4.83E-16	ERBB2	2.119	4.07E-13	HNF1B	-3.317	1.65E-14
NFkB (complex)	2.885	4.96E-16	HGF	2.201	1.13E-12	IL1B	2.641	7.61E-14
TGFb3	3.515	9.63E-16	HNF1B	-3.162	1.71E-12	JUN	2.908	1.08E-13
HNF1B	-3.742	1.15E-15	CTNNB1	2.953	6.91E-11	SP1	2.438	8.66E-12

### Platelets induce similar changes in cancer animal models and human cell lines

**Fig.5:**
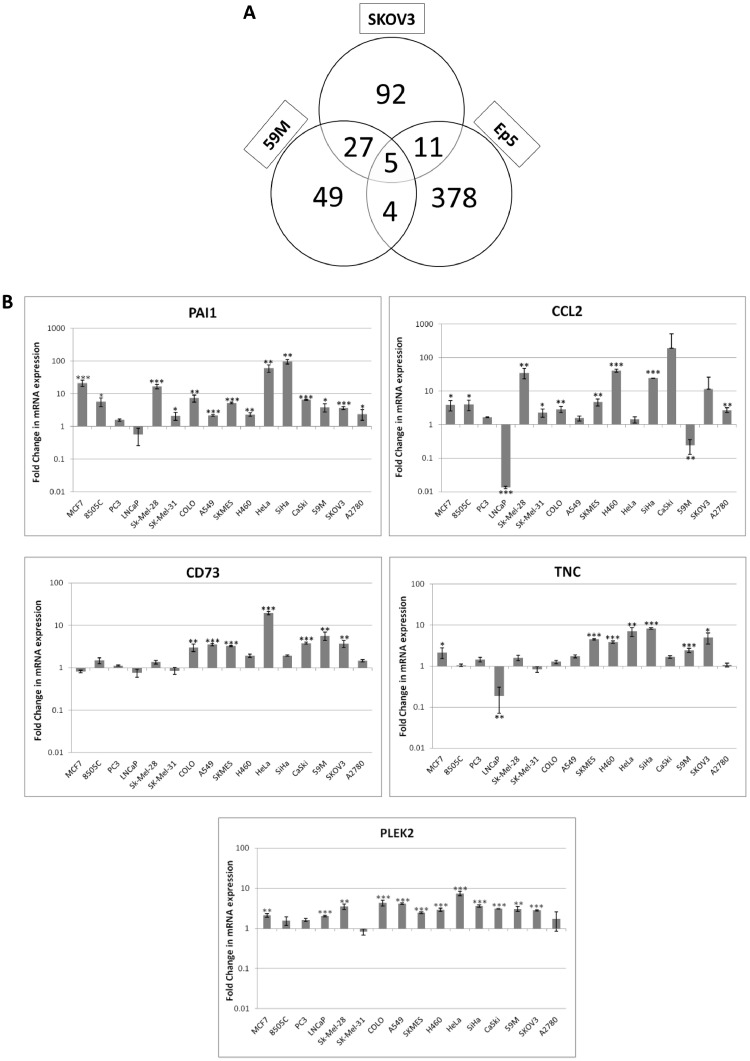
Platelets induce similar changes in animal and human models, a panel of 5 genes were identified as key mediators in this process. (A) Venn Diagram depicting the commonly dysregulated genes between the two ovarian models (SKOV3 and 59M) and the mouse model (Ep5) [22]. (B) PAI-1, PLEK2, CD73 and TNC and CCL2 were validated in a panel of 16 cancer cell lines using a custom panel on the TaqMan OpenArray® platform. Data shown are mean values ± SD (*n* = 3) and are expressed as fold change in mRNA expression in cancer cells co-cultured in the presence of platelets relative to control cells grown in the absence of platelets (for 24 h). Values are normalised to the endogenous control. *** *p* ≤ 0.001, ** *p* ≤ 0.01, * *p* ≤ 0.05.

Labelle and colleagues [23] examined the effect of platelets on gene expression levels in a mouse breast cancer cell line (Ep5). To identify the potential key drivers of the interaction between platelets and cancer cells, a comparison was made between the mouse and human gene expression profiles. A panel of 5 genes commonly dysregulated in the two ovarian models described here and the mouse model (Ep5) were identified (Fig. 5); comprised of 4 up-regulated genes, plasminogen activator inhibitor 1 (PAI-1), Pleckstrin-2 (PLEK2), cluster of differentiation 73 (CD73) and Tenascin C (TNC), and one down-regulated gene, serum deprivation-response protein (SDPR). Interestingly, we identified a further gene monocyte chemoattractant Protein-1 (MCP-1/CCL2), which is a known chemoattractant for monocytes and basophils, which was significantly up-regulated in the SKOV3 and Ep5 cell lines but significantly down-regulated in the 59M cell line.

The up-regulated genes and CCL2, were validated on a panel of 16 cell lines. The prostate cancer cell line, LNCaP, was also included to determine if the prostate observations derived using the PC-3 model were cell line specific. With the exception of the prostate cancer cell lines PC-3 and LNCaP, all of the cell lines demonstrated a significant increase in the expression of the mesenchymal marker PAI-1. PLEK2 was also broadly upregulated with significant increases in expression evident in 80% (12/16) of the cell lines tested. CD73 and TNC were significantly upregulated in 7 of the cell lines, with CCL2 expression significantly increased in 9 of the cell lines.

## Discussion

We and others have previously shown that the platelet-cancer cell interactions, also known as ‘platelet cloaks’, provide an advantage to cancer cells during metastasis in numerous cancer models [7,24]. Having previously shown this phenomenon in ovarian cancer [7], here we demonstrate the induction of a mesenchymal-like phenotype by platelet cloaks to be a universal phenomenon in cancer cells. Platelets were found to interact similarly with all of the cancer cell types tested whilst also displaying cancer cell and cell-line specific heterogeneity in their levels of adherence and activation and, with the exception of prostate cancer cells, their imprint on cancer cell gene expression. The variable platelet binding and activation may depend on the metastatic potential of the cancer cell lines or how the different cancer cell lines respond to EMT signals, with cell lines like PC-3 already displaying a migratory spindle shaped phenotype. As EMT is an essential component during the initiation of metastasis, understanding this mechanism is a possible avenue to finding treatment or preventative strategies targeting metastasis. To further understand this mechanism, a whole gene transcriptome analysis of platelet-cloaked cancer cells was performed, which identified a 34-gene signature that we believe to encompass key mediators of cancer cell-platelet interaction. Our work reveals a new elucidation of the platelet cloak mechanism through which EMT can be induced/enhanced and an associated gene signature containing potential therapeutic targets through which metastasis may be impaired.

The ability of platelets to induce a metastatic phenotype was previously thought to be a contribution of their adhesion capacity and their ability to form a cloak or shield around tumour cells [25,26], thus protecting them from NK cell mediated cytotoxicity, shear stress and enabling endothelial adhesion [5,[27], [28], [29], [30]]. However, it is now increasingly evident that platelets are capable of imparting upon tumour cells by more complex signalling mechanisms to increase their metastatic potential. The induction of EMT and/or maintenance of EMT is one such mechanism. While our studies across 7 cancer cell models demonstrate the universal ability of platelets to interact with cancer cells, our results also corroborate what others have found in specific cancer types or systems [23,31,32]. The promotion of EMT by platelets demonstrated by changes in cancer cell gene expression and phenotype, and increases in their invasion capacity, leads to a more aggressive metastatic phenotype. In colorectal cancer, the cancer cell platelet interaction was found to activate the Wnt-β-catenin pathway, leading to expression of an aggressive EMT phenotype [33]. Similarly, in breast cancer, the accumulation of platelets has been shown to induce EMT and promote chemoresistance [34]. Despite displaying a reduced molecular signature in response to the platelet interaction, the PC-3 cell line did display increased invasion suggesting other genes may be involved in this response. While others have shown the ability of PDGF to induce EMT in the PC-3 model [35], further work is needed to clarify the reduced molecular signature in response to platelet interaction. This might be explained by a higher propensity to cloak with other cell types like macrophages or neutrophils. A recent study has shown that prostate cell line models can vary in their ability to induce platelet aggregation which is linked to their androgen receptor expression and this may also explain the reciprocal trends observed between the PC-3 and LNCaP [36] that represent androgen independent and dependent models respectively.

Platelets contain high concentrations of TGFβ [5,30] and it is therefore not surprising to find that the 34 gene panel identified in this study are either regulated by or directly regulate TGFβ. This is also in agreement with previous studies showing the induction of the pro-metastatic phenotype *via* activation of the TGFβ signalling pathway in different cancer systems [37,38]. Platelet-derived α-granules, containing TGFβ have been demonstrated to increase the growth of primary tumours in ovarian mouse models [39,40], while inhibiting TGFβ signalling in the tumour cell or blocking its expression in platelets has been shown to inhibit metastasis and EMT [23]. We and others have previously demonstrated that both direct contact and the platelet releasate are important factors in the platelet-cancer cell interaction [7,23]. In breast cancer, integrin α2β1 has been shown to mediate the direct interaction between platelet and MCF-7 cells, promoting EMT and invasion of MCF-7 cells [41]. While we did not see a significant increase in invasion in the MCF-7 line, we did observe a positive trend. The observations reported suggest a wider role of TGFβ in bringing about EMT in different cancers, as well as the universal nature of this interaction.

The TGFβ pathway has important implications in the clinical setting; platelets are providing a source of TGFβ to the circulating tumour cells and driving a metastatic phenotype at the site of intravasation and extravasation. Targeting the key molecules involved in this interaction has the potential to inhibit the development of this metastatic phenotype. The further refinement of this panel and identification of the 5 overlapping genes in both human and mouse ovarian cancer models strengthens the evidence for the key role of these genes in the cancer cell platelet interaction and their potential as therapeutic targets in the clinical setting. Individually, these genes have been shown to play a role in metastasis. PAI-1, one of the top dysregulated genes in this panel, was further validated across the panel of cancer cell lines and found to be upregulated in 12 of the 16 cell lines, the prostate cancer cell lines (PC-3 and LNCaP) being the exception, which may be related to the role of human kallikrein 2 which has been shown to inactivate PAI-1 in prostate cancer [42]. PAI-1 is a serine protease, which functions as an inhibitor of tissue plasminogen activator and urokinase plasminogen activator. While platelets are known to contain high levels of PAI-1, the expression was negligible with the concentration of platelets used in this study as demonstrated by our microarray analysis, which did not identify PAI-1 as a specific platelet mRNA contributing to our gene signatures. PAI-1 is a known mesenchymal marker confirming the phenotypic and genotypic patterns observed here following the platelet - cancer cell interaction. PAI-1 is expressed in many solid tumours and is associated with a poor prognosis and chemoresistance [43]. The role of PAI-1 in metastasis is conflicting with some studies displaying a pro-metastatic impact and others an inhibitory role. In addition, no inhibitors of PAI-1 have shown clinical effectiveness. Further work is needed to fully interrogate the role of PAI-1 and to determine if the presence of the platelet is necessary for the associated pro-metastatic effect. The evidence for PLEK2 in metastasis is just emerging and closely linked with TGFβ signalling in lung cancer [44] and EGFR signalling in bladder cancer [45]. CD73, expressed in many tumour types and immune cells, has been associated with a metastatic phenotype in melanoma and breast cancer [46,47]. Antibody therapies to CD73 are ongoing in many clinical trials with many studies now combining this with checkpoint inhibitors (NCT04148937) (www.clinicaltrials.gov). TNC is a glycoprotein of the ECM and frequently associated with cancer, including metastasis initiation and EMT [48,49]. Further work is needed however to fully understand its role in metastasis. CCL2 is a chemokine and a chemotactic factor for monocytes and had been reported to be increased in many cancer types [50]. Many clinical trials are ongoing with inhibitors of CCL2, but results have been disappointing to date. Further understanding of this molecule is needed to fully exploit its role in metastasis. While these genes seem to be key mediators in the cancer cell-platelet interaction and appear to have essential roles in cancer and metastasis, it not fully understood if a combination approach is needed to target these essential molecules. Further work is needed to fully understand this.

In summary, platelet-adhesion to cancer cells appears to be a universal process, resulting in an EMT-driven phenotype. This process is a key factor in the pro-metastatic phenotype and is mediated by 5 key genes which have established roles in metastasis. A better understanding of the role of the platelets in driving a pro-metastatic phenotype through these key mediators is needed in order to target metastasis and improve outcomes for cancer patients.

## CRediT authorship contribution statement

**Cathy D. Spillane:** Conceptualization, Methodology, Validation, Formal analysis, Investigation, Data curation, Writing – original draft, Writing – review & editing, Visualization, Project administration, Funding acquisition. **Niamh M. Cooke:** Conceptualization, Methodology, Validation, Formal analysis, Investigation, Data curation, Writing – review & editing. **Mark P. Ward:** Writing – original draft, Writing – review & editing, Visualization. **Dermot Kenny:** Conceptualization, Methodology, Writing – review & editing, Supervision. **Gordon Blackshields:** Software, Formal analysis, Resources. **Tanya Kelly:** Writing – original draft, Writing – review & editing. **Mark Bates:** Writing – original draft. **Yanmei Huang:** Writing – original draft, Writing – review & editing, Visualization. **Cara Martin:** Writing – review & editing. **Sinead Skehan:** Investigation, Writing – review & editing. **Aoife Canney:** Investigation, Writing – review & editing. **Michael Gallagher:** Writing – original draft, Writing – review & editing. **Paul Smyth:** Methodology, Investigation. **Nathan Brady:** Writing – review & editing. **Andres Clarke:** Investigation, Writing – review & editing. **Bashir Mohamed:** Writing – review & editing. **Lucy Norris:** Writing – review & editing. **Doug A. Brooks:** Writing – review & editing, Visualization. **Robert D. Brooks:** Writing – review & editing, Visualization. **Jessica K. Heatlie:** . **Stavros Selemidis:** Investigation, Writing – review & editing. **Sean Hanniffy:** Writing – review & editing. **Eric Dixon:** Writing – review & editing. **Orla Sheils:** Conceptualization, Methodology, Writing – review & editing, Project administration, Supervision, Funding acquisition. **Sharon A. O'Toole:** Writing – original draft, Writing – review & editing, Visualization, Supervision, Project administration. **John J. O'Leary:** Conceptualization, Methodology, Writing – original draft, Writing – review & editing, Visualization, Supervision, Project administration, Funding acquisition.

## Declaration of Competing Interest

The authors have declared that no competing interests exists.

## Appendix Supplementary materials

Image, application 1
