# Supplementary material for: The induction of a mesenchymal phenotype by platelet cloaking of cancer cells is a universal phenomenon
Source: Transl Oncol. 2021 Sep 27;14(12):101229. doi: 10.1016/j.tranon.2021.101229 (PMC8488306; doi:10.1016/j.tranon.2021.101229)
Supplement: Supplementary file 1 [file mmc1.docx]

**Supplementary Tables and Figures**

**Supplementary Table 1: Cell culture media source and relevant supplements**

| **Cell Line** | **Cancer type** | **Source** | **Growth media** | **Additional supplements** |
| --- | --- | --- | --- | --- |
| PC3 | Prostate | ATCC | Ham’s F-12K |  |
| MCF7 | Breast | ECACC | EMEM | 1X NEAA |
| 8505C | Thyroid | ECACC | EMEM | 1X NEAA |
| SK-Mel-28 | Melanoma | ATCC | EMEM |  |
| SK-Mel-31 | Melanoma | ATCC | EMEM | 15% FBS, 1X NEAA |
| COLO794 | Melanoma | ECACC | RPMI 1640 |  |
| A549 | Lung | ATCC | Ham’s F-12K |  |
| SKMES1 | Lung | ATCC | EMEM | 1X NEAA |
| H460 | Lung | ATCC | RPMI 1640 |  |
| HeLa | Cervical | ATCC | EMEM | 1X NEAA |
| SiHa | Cervical | ATCC | EMEM | 1X NEAA |
| CaSki | Cervical | ATCC | RPMI 1640 |  |
| 59M | Ovarian | ECACC | DMEM |  |
| SKOV3 | Ovarian | ATCC | McCoy’s |  |
| A2780 | Ovarian | ECACC | RPMI 1640 |  |

NEAA = non-essential amino acids

Primary cell lines: 8505C, SiHa, A2780, A549 and SKMel28

**Supplementary Table 2: Significantly^1^ altered gene expression in SKOV3 and 59M cells after 24 hrs co-culture with platelets.**

| **Expression in:** | | **SKOV3** | | **59M** | |
| --- | --- | --- | --- | --- | --- |
| **Symbol** | **Entrez** | **FC** | **FDR** | **FC** | **FDR** |
| [LAMC2](http://www.ncbi.nlm.nih.gov/gene/?term=LAMC2) | 3918 | 7.8 | 4.4E-12 | 5.8 | 7.21E-05 |
| [MMP2](http://www.ncbi.nlm.nih.gov/gene/?term=MMP2) | 4313 | 2.4 | 6.6E-05 | 3.0 | 1.29E-04 |
| [PMEPA1](http://www.ncbi.nlm.nih.gov/gene/?term=PMEPA1) | 56937 | 2.7 | 7.5E-06 | 3.0 | 2.72E-04 |
| [NT5E](http://www.ncbi.nlm.nih.gov/gene/?term=NT5E) | 4907 | 2.3 | 4.7E-05 | 2.9 | 3.02E-04 |
| [RP11-597K23.2](http://www.ncbi.nlm.nih.gov/gene/?term=RP11-597K23.2) | 101929690 | 3.0 | 2.0E-05 | 2.9 | 3.02E-04 |
| [TFPI2](http://www.ncbi.nlm.nih.gov/gene/?term=TFPI2) | 7980 | 2.8 | 1.5E-06 | 2.8 | 6.94E-04 |
| [MMP1](http://www.ncbi.nlm.nih.gov/gene/?term=MMP1) | 4312 | 1.9 | 3.6E-03 | 2.7 | 4.67E-03 |
| [DOCK4](http://www.ncbi.nlm.nih.gov/gene/?term=DOCK4) | 9732 | 1.8 | 8.7E-03 | 2.6 | 2.72E-04 |
| [SEMA7A](http://www.ncbi.nlm.nih.gov/gene/?term=SEMA7A) | 8482 | 2.4 | 1.8E-04 | 2.5 | 2.07E-03 |
| [G0S2](http://www.ncbi.nlm.nih.gov/gene/?term=G0S2) | 50486 | 2.3 | 6.5E-05 | 2.3 | 8.29E-03 |
| [NRP2](http://www.ncbi.nlm.nih.gov/gene/?term=NRP2) | 8828 | 2.0 | 8.2E-04 | 2.3 | 7.73E-03 |
| [PLAUR](http://www.ncbi.nlm.nih.gov/gene/?term=PLAUR) | 5329 | 2.7 | 2.1E-06 | 2.3 | 6.94E-04 |
| [TGFBI](http://www.ncbi.nlm.nih.gov/gene/?term=TGFBI) | 7045 | 2.2 | 2.4E-04 | 2.2 | 6.94E-04 |
| [ADAM19](http://www.ncbi.nlm.nih.gov/gene/?term=ADAM19) | 8728 | 4.7 | 1.9E-09 | 2.2 | 2.60E-03 |
| [DNER](http://www.ncbi.nlm.nih.gov/gene/?term=DNER) | 92737 | 2.2 | 3.5E-04 | 2.2 | 1.43E-03 |
| [SERPINE1](http://www.ncbi.nlm.nih.gov/gene/?term=SERPINE1) | 5054 | 2.7 | 6.1E-06 | 2.2 | 7.27E-04 |
| [EPHB2](http://www.ncbi.nlm.nih.gov/gene/?term=EPHB2) | 2048 | 2.2 | 8.6E-05 | 2.1 | 6.94E-04 |
| [LAMB3](http://www.ncbi.nlm.nih.gov/gene/?term=LAMB3) | 3914 | 3.2 | 4.6E-07 | 2.0 | 1.56E-02 |
| [HIC1](http://www.ncbi.nlm.nih.gov/gene/?term=HIC1) | 3090 | 1.8 | 1.9E-02 | 1.9 | 1.49E-02 |
| [TNC](http://www.ncbi.nlm.nih.gov/gene/?term=TNC) | 3371 | 3.1 | 9.2E-07 | 1.9 | 1.88E-03 |
| [PLEK2](http://www.ncbi.nlm.nih.gov/gene/?term=PLEK2) | 26499 | 2.6 | 8.3E-06 | 1.9 | 2.26E-02 |
| [PLAU](http://www.ncbi.nlm.nih.gov/gene/?term=PLAU) | 5328 | 2.3 | 5.9E-05 | 1.8 | 1.60E-02 |
| [ANPEP](http://www.ncbi.nlm.nih.gov/gene/?term=ANPEP) | 290 | 1.9 | 3.3E-03 | 1.8 | 5.94E-03 |
| [SEMA3C](http://www.ncbi.nlm.nih.gov/gene/?term=SEMA3C) | 10512 | 1.8 | 3.9E-03 | 1.8 | 2.04E-02 |
| [PTGIS](http://www.ncbi.nlm.nih.gov/gene/?term=PTGIS) | 5740 | -3.2 | 5.3E-07 | -1.8 | 1.67E-02 |
| [EGR1](http://www.ncbi.nlm.nih.gov/gene/?term=EGR1) | 1958 | -1.9 | 7.6E-03 | -1.8 | 8.29E-03 |
| [ID1](http://www.ncbi.nlm.nih.gov/gene/?term=ID1) | 3397 | -2.3 | 3.5E-04 | -1.9 | 5.39E-03 |
| [SDPR](http://www.ncbi.nlm.nih.gov/gene/?term=SDPR) | 8436 | -2.2 | 1.4E-04 | -1.9 | 3.46E-03 |
| [CITED2](http://www.ncbi.nlm.nih.gov/gene/?term=CITED2) | 10370 | -3.2 | 4.6E-07 | -1.9 | 6.35E-03 |
| [PEG10](http://www.ncbi.nlm.nih.gov/gene/?term=PEG10) | 23089 | -2.5 | 1.4E-04 | -2.0 | 6.94E-04 |
| [NQO1](http://www.ncbi.nlm.nih.gov/gene/?term=NQO1) | 1728 | -1.8 | 6.1E-03 | -2.1 | 1.06E-02 |
| [CPA4](http://www.ncbi.nlm.nih.gov/gene/?term=CPA4) | 51200 | -1.8 | 4.3E-03 | -2.3 | 6.94E-04 |
| [ITGB8](http://www.ncbi.nlm.nih.gov/gene/?term=ITGB8) | 3696 | -2.3 | 6.9E-05 | -3.0 | 3.04E-04 |
| [CYP1B1](http://www.ncbi.nlm.nih.gov/gene/?term=CYP1B1) | 1545 | -3.5 | 2.6E-07 | -3.2 | 2.72E-04 |
| [IL8](http://www.ncbi.nlm.nih.gov/gene/?term=IL8) | 3576 | 3.6 | 3.0E-05 | 1.97 | 0.12 |
| [PDCD1LG2](http://www.ncbi.nlm.nih.gov/gene/?term=PDCD1LG2) | 80380 | 2.2 | 1.3E-04 | 1.74 | 0.08 |
| [ANGPTL4](http://www.ncbi.nlm.nih.gov/gene/?term=ANGPTL4) | 51129 | 2.6 | 1.7E-05 | 1.73 | 0.01 |
| [LAMB1](http://www.ncbi.nlm.nih.gov/gene/?term=LAMB1) | 3912 | 1.9 | 1.4E-03 | 1.73 | 0.01 |
| [ADAMTS6](http://www.ncbi.nlm.nih.gov/gene/?term=ADAMTS6) | 11174 | 2.2 | 1.4E-04 | 1.72 | 0.06 |
| [LTBP2](http://www.ncbi.nlm.nih.gov/gene/?term=LTBP2) | 4053 | 1.9 | 3.3E-03 | 1.71 | 0.01 |
| [ITGA2](http://www.ncbi.nlm.nih.gov/gene/?term=ITGA2) | 3673 | 2.3 | 7.6E-05 | 1.71 | 0.02 |
| [SPOCK1](http://www.ncbi.nlm.nih.gov/gene/?term=SPOCK1) | 6695 | 2.4 | 3.6E-05 | 1.66 | 0.02 |
| [ADAM12](http://www.ncbi.nlm.nih.gov/gene/?term=ADAM12) | 8038 | 1.9 | 9.4E-03 | 1.63 | 0.27 |
| [SPHK1](http://www.ncbi.nlm.nih.gov/gene/?term=SPHK1) | 8877 | 2.4 | 5.9E-05 | 1.63 | 0.03 |
| [ANTXR2](http://www.ncbi.nlm.nih.gov/gene/?term=ANTXR2) | 118429 | 2.3 | 5.9E-05 | 1.61 | 0.03 |
| [FRMD6](http://www.ncbi.nlm.nih.gov/gene/?term=FRMD6) | 122786 | 2.2 | 3.5E-04 | 1.57 | 0.07 |
| [SMURF2](http://www.ncbi.nlm.nih.gov/gene/?term=SMURF2) | 64750 | 1.9 | 1.4E-03 | 1.57 | 0.08 |
| [ELK3](http://www.ncbi.nlm.nih.gov/gene/?term=ELK3) | 2004 | 1.9 | 3.7E-03 | 1.56 | 0.09 |
| [SLC22A4](http://www.ncbi.nlm.nih.gov/gene/?term=SLC22A4) | 6583 | 1.8 | 2.1E-02 | 1.54 | 0.04 |
| [LPCAT2](http://www.ncbi.nlm.nih.gov/gene/?term=LPCAT2) | 54947 | 1.8 | 7.1E-03 | 1.54 | 0.06 |
| [IL6](http://www.ncbi.nlm.nih.gov/gene/?term=IL6) | 3569 | 2.2 | 1.5E-04 | 1.51 | 0.33 |
| [MAL](http://www.ncbi.nlm.nih.gov/gene/?term=MAL) | 4118 | -3.8 | 1.6E-08 | -1.53 | 0.11 |
| [QPRT](http://www.ncbi.nlm.nih.gov/gene/?term=QPRT) | 101929880 | -1.8 | 2.5E-02 | -1.54 | 0.17 |
| [ITGB4](http://www.ncbi.nlm.nih.gov/gene/?term=ITGB4) | 3691 | -2.5 | 6.6E-05 | -1.61 | 0.03 |
| [ZFP36](http://www.ncbi.nlm.nih.gov/gene/?term=ZFP36) | 7538 | -2.5 | 3.3E-05 | -1.83 | 0.07 |
| podocalyxin-like | 5420 | 1.72 | 0.01 | 2.6 | 2.7E-04 |
| CUB domain containing protein 1 | 64866 | 1.71 | 0.01 | 2.0 | 8.3E-03 |
| interleukin 11 | 3589 | 1.71 | 0.02 | 1.9 | 1.3E-02 |
| neuron navigator 3 | 89795 | 1.68 | 0.02 | 1.8 | 7.7E-03 |
| vascular endothelial growth factor C | 7424 | 1.65 | 0.03 | 1.8 | 2.0E-02 |
| growth differentiation factor 6 | 392255 | 1.65 | 0.10 | 1.8 | 9.3E-03 |
| cyclin-dependent kinase 17 | 5128 | 1.64 | 0.02 | 1.8 | 3.7E-02 |
| adhesion molecule with Ig-like domain 2 | 347902 | 1.61 | 0.04 | 2.2 | 7.7E-04 |
| abhydrolase domain containing 2 | 11057 | 1.60 | 0.03 | 2.0 | 1.3E-03 |
| sialic acid binding Ig-like lectin 15 | 284266 | 1.59 | 0.04 | 2.4 | 2.7E-04 |
| latent transforming growth factor beta binding protein 1 | 4052 | 1.53 | 0.06 | 1.8 | 6.4E-03 |
| protein tyrosine phosphatase, receptor type, K | 5796 | 1.52 | 0.07 | 1.9 | 7.2E-03 |
| cyclin D1 | 595 | 1.51 | 0.07 | 1.8 | 1.2E-02 |
| epoxide hydrolase 1, microsomal (xenobiotic) | 2052 | -1.49 | 0.08 | -1.9 | 2.8E-02 |
| Rho-related BTB domain containing 3 | 22836 | -1.50 | 0.10 | -1.9 | 5.9E-03 |
| occludin | 100506658 | -1.52 | 0.20 | -1.9 | 4.6E-03 |
| CD70 molecule | 970 | -1.57 | 0.08 | -1.9 | 2.5E-03 |
| v-kit Hardy-Zuckerman 4 feline sarcoma viral oncogene homolog | 3815 | -1.64 | 0.04 | -1.9 | 1.3E-02 |
| [EDNRA](http://www.ncbi.nlm.nih.gov/gene/?term=EDNRA) | 1909 | 4.7 | 1.3E-08 | - | - |
| [CALB2](http://www.ncbi.nlm.nih.gov/gene/?term=CALB2) | 794 | 4.4 | 4.5E-09 | - | - |
| [FERMT1](http://www.ncbi.nlm.nih.gov/gene/?term=FERMT1) | 55612 | 2.9 | 2.5E-06 | - | - |
| [SRPX](http://www.ncbi.nlm.nih.gov/gene/?term=SRPX) | 8406 | 2.8 | 1.7E-05 | - | - |
| [ITGA5](http://www.ncbi.nlm.nih.gov/gene/?term=ITGA5) | 3678 | 2.6 | 6.9E-06 | - | - |
| [XDH](http://www.ncbi.nlm.nih.gov/gene/?term=XDH) | 7498 | 2.6 | 2.0E-05 | - | - |
| [JAG1](http://www.ncbi.nlm.nih.gov/gene/?term=JAG1) | 182 | 2.5 | 8.3E-06 | - | - |
| [TNFAIP3](http://www.ncbi.nlm.nih.gov/gene/?term=TNFAIP3) | 7128 | 2.5 | 2.8E-02 | - | - |
| [SLC22A3](http://www.ncbi.nlm.nih.gov/gene/?term=SLC22A3) | 6581 | 2.5 | 4.8E-05 | - | - |
| [GPAM](http://www.ncbi.nlm.nih.gov/gene/?term=GPAM) | 57678 | 2.5 | 6.3E-05 | - | - |
| [LMCD1](http://www.ncbi.nlm.nih.gov/gene/?term=LMCD1) | 29995 | 2.4 | 3.6E-05 | - | - |
| [FGD6](http://www.ncbi.nlm.nih.gov/gene/?term=FGD6) | 55785 | 2.4 | 8.9E-05 | - | - |
| [MBOAT2](http://www.ncbi.nlm.nih.gov/gene/?term=MBOAT2) | 129642 | 2.3 | 4.6E-05 | - | - |
| [ZNF185](http://www.ncbi.nlm.nih.gov/gene/?term=ZNF185) | 7739 | 2.2 | 1.1E-04 | - | - |
| [CDH6](http://www.ncbi.nlm.nih.gov/gene/?term=CDH6) | 1004 | 2.2 | 1.8E-04 | - | - |
| [COL4A1](http://www.ncbi.nlm.nih.gov/gene/?term=COL4A1) | 1282 | 2.1 | 2.3E-04 | - | - |
| [TNFAIP6](http://www.ncbi.nlm.nih.gov/gene/?term=TNFAIP6) | 7130 | 2.1 | 1.8E-02 | - | - |
| [PTHLH](http://www.ncbi.nlm.nih.gov/gene/?term=PTHLH) | 5744 | 2.1 | 2.4E-04 | - | - |
| [GLIPR1](http://www.ncbi.nlm.nih.gov/gene/?term=GLIPR1) | 11010 | 2.1 | 4.1E-04 | - | - |
| [ESM1](http://www.ncbi.nlm.nih.gov/gene/?term=ESM1) | 11082 | 2.1 | 2.3E-04 | - | - |
| [C3orf52](http://www.ncbi.nlm.nih.gov/gene/?term=C3orf52) | 79669 | 2.1 | 4.8E-04 | - | - |
| [NET1](http://www.ncbi.nlm.nih.gov/gene/?term=NET1) | 10276 | 2.1 | 4.8E-04 | - | - |
| [CTGF](http://www.ncbi.nlm.nih.gov/gene/?term=CTGF) | 1490 | 2.1 | 2.8E-04 | - | - |
| [E2F7](http://www.ncbi.nlm.nih.gov/gene/?term=E2F7) | 144455 | 2.1 | 4.9E-04 | - | - |
| [PLXNA2](http://www.ncbi.nlm.nih.gov/gene/?term=PLXNA2) | 5362 | 2.0 | 5.8E-04 | - | - |
| [SLFN5](http://www.ncbi.nlm.nih.gov/gene/?term=SLFN5) | 162394 | 2.0 | 4.1E-04 | - | - |
| [GNPNAT1](http://www.ncbi.nlm.nih.gov/gene/?term=GNPNAT1) | 64841 | 2.0 | 1.9E-03 | - | - |
| [MYH8](http://www.ncbi.nlm.nih.gov/gene/?term=MYH8) | 4626 | 2.0 | 1.9E-03 | - | - |
| [TNS1](http://www.ncbi.nlm.nih.gov/gene/?term=TNS1) | 7145 | 1.9 | 1.2E-03 | - | - |
| [SOX4](http://www.ncbi.nlm.nih.gov/gene/?term=SOX4) | 6659 | 1.9 | 1.1E-03 | - | - |
| [PLAT](http://www.ncbi.nlm.nih.gov/gene/?term=PLAT) | 5327 | 1.9 | 3.6E-03 | - | - |
| [ITGA6](http://www.ncbi.nlm.nih.gov/gene/?term=ITGA6) | 3655 | 1.9 | 1.2E-03 | - | - |
| [CHST11](http://www.ncbi.nlm.nih.gov/gene/?term=CHST11) | 50515 | 1.9 | 1.6E-03 | - | - |
| [NFKBIA](http://www.ncbi.nlm.nih.gov/gene/?term=NFKBIA) | 4792 | 1.9 | 2.0E-02 | - | - |
| [FLRT3](http://www.ncbi.nlm.nih.gov/gene/?term=FLRT3) | 23767 | 1.9 | 3.9E-03 | - | - |
| [HAS2](http://www.ncbi.nlm.nih.gov/gene/?term=HAS2) | 3037 | 1.9 | 6.3E-03 | - | - |
| [SLC16A9](http://www.ncbi.nlm.nih.gov/gene/?term=SLC16A9) | 220963 | 1.9 | 2.3E-03 | - | - |
| [ITGAV](http://www.ncbi.nlm.nih.gov/gene/?term=ITGAV) | 3685 | 1.8 | 4.2E-03 | - | - |
| [SFR1](http://www.ncbi.nlm.nih.gov/gene/?term=SFR1) | 119392 | 1.8 | 5.1E-03 | - | - |
| [LRRC38](http://www.ncbi.nlm.nih.gov/gene/?term=LRRC38) | 126755 | 1.8 | 5.5E-03 | - | - |
| [P2RY2](http://www.ncbi.nlm.nih.gov/gene/?term=P2RY2) | 5029 | 1.8 | 4.4E-03 | - | - |
| [CD59](http://www.ncbi.nlm.nih.gov/gene/?term=CD59) | 966 | 1.8 | 4.3E-03 | - | - |
| [HIST1H2AB](http://www.ncbi.nlm.nih.gov/gene/?term=HIST1H2AB) | 8335 | 1.8 | 5.0E-02 | - | - |
| [EFR3B](http://www.ncbi.nlm.nih.gov/gene/?term=EFR3B) | 22979 | 1.8 | 8.3E-03 | - | - |
| [JUNB](http://www.ncbi.nlm.nih.gov/gene/?term=JUNB) | 3726 | 1.8 | 8.2E-03 | - | - |
| [TMC7](http://www.ncbi.nlm.nih.gov/gene/?term=TMC7) | 79905 | 1.8 | 9.1E-03 | - | - |
| [CMTM3](http://www.ncbi.nlm.nih.gov/gene/?term=CMTM3) | 123920 | 1.8 | 1.4E-02 | - | - |
| [MMP10](http://www.ncbi.nlm.nih.gov/gene/?term=MMP10) | 4319 | 1.8 | 2.2E-02 | - | - |
| [HBEGF](http://www.ncbi.nlm.nih.gov/gene/?term=HBEGF) | 1839 | 1.8 | 1.8E-02 | - | - |
| [IL13RA2](http://www.ncbi.nlm.nih.gov/gene/?term=IL13RA2) | 3598 | 1.8 | 1.2E-02 | - | - |
| [NGF](http://www.ncbi.nlm.nih.gov/gene/?term=NGF) | 4803 | 1.8 | 1.2E-02 | - | - |
| [BVES](http://www.ncbi.nlm.nih.gov/gene/?term=BVES) | 11149 | 1.8 | 1.4E-02 | - | - |
| [CDH16](http://www.ncbi.nlm.nih.gov/gene/?term=CDH16) | 1014 | -1.8 | 1.9E-02 | - | - |
| [LRRN4](http://www.ncbi.nlm.nih.gov/gene/?term=LRRN4) | 164312 | -1.8 | 3.3E-02 | - | - |
| [CYP24A1](http://www.ncbi.nlm.nih.gov/gene/?term=CYP24A1) | 1591 | -1.8 | 9.8E-03 | - | - |
| [PIK3R1](http://www.ncbi.nlm.nih.gov/gene/?term=PIK3R1) | 5295 | -1.8 | 2.3E-02 | - | - |
| [DLX3](http://www.ncbi.nlm.nih.gov/gene/?term=DLX3) | 1747 | -1.8 | 9.8E-03 | - | - |
| [ANXA4](http://www.ncbi.nlm.nih.gov/gene/?term=ANXA4) | 307 | -1.8 | 3.8E-03 | - | - |
| [FGFBP1](http://www.ncbi.nlm.nih.gov/gene/?term=FGFBP1) | 9982 | -1.8 | 1.0E-02 | - | - |
| [ZIC2](http://www.ncbi.nlm.nih.gov/gene/?term=ZIC2) | 7546 | -1.8 | 5.7E-03 | - | - |
| [CCNA1](http://www.ncbi.nlm.nih.gov/gene/?term=CCNA1) | 8900 | -1.8 | 3.1E-03 | - | - |
| [ARID5B](http://www.ncbi.nlm.nih.gov/gene/?term=ARID5B) | 84159 | -1.9 | 8.6E-03 | - | - |
| [DCDC2](http://www.ncbi.nlm.nih.gov/gene/?term=DCDC2) | 51473 | -1.9 | 3.7E-03 | - | - |
| [PLEKHA7](http://www.ncbi.nlm.nih.gov/gene/?term=PLEKHA7) | 144100 | -1.9 | 1.6E-03 | - | - |
| [TMEM47](http://www.ncbi.nlm.nih.gov/gene/?term=TMEM47) | 83604 | -1.9 | 2.2E-03 | - | - |
| [HEXIM1](http://www.ncbi.nlm.nih.gov/gene/?term=HEXIM1) | 10614 | -1.9 | 3.0E-03 | - | - |
| [SLC4A11](http://www.ncbi.nlm.nih.gov/gene/?term=SLC4A11) | 83959 | -1.9 | 1.4E-03 | - | - |
| [ANKRD1](http://www.ncbi.nlm.nih.gov/gene/?term=ANKRD1) | 27063 | -2.0 | 2.0E-03 | - | - |
| [SLC7A11](http://www.ncbi.nlm.nih.gov/gene/?term=SLC7A11) | 23657 | -2.0 | 7.1E-04 | - | - |
| [KRT80](http://www.ncbi.nlm.nih.gov/gene/?term=KRT80) | 144501 | -2.0 | 1.1E-02 | - | - |
| [ALDH3A1](http://www.ncbi.nlm.nih.gov/gene/?term=ALDH3A1) | 218 | -2.0 | 7.1E-04 | - | - |
| [DUSP1](http://www.ncbi.nlm.nih.gov/gene/?term=DUSP1) | 1843 | -2.0 | 4.7E-03 | - | - |
| [NR4A1](http://www.ncbi.nlm.nih.gov/gene/?term=NR4A1) | 3164 | -2.0 | 5.8E-04 | - | - |
| [DPP4](http://www.ncbi.nlm.nih.gov/gene/?term=DPP4) | 1803 | -2.1 | 4.0E-04 | - | - |
| [IDO1](http://www.ncbi.nlm.nih.gov/gene/?term=IDO1) | 3620 | -2.1 | 7.1E-03 | - | - |
| [SMAD6](http://www.ncbi.nlm.nih.gov/gene/?term=SMAD6) | 4091 | -2.1 | 3.5E-04 | - | - |
| [ID3](http://www.ncbi.nlm.nih.gov/gene/?term=ID3) | 3399 | -2.2 | 6.0E-04 | - | - |
| [FOS](http://www.ncbi.nlm.nih.gov/gene/?term=FOS) | 2353 | -2.2 | 3.4E-03 | - | - |
| [CTH](http://www.ncbi.nlm.nih.gov/gene/?term=CTH) | 1491 | -2.2 | 2.3E-04 | - | - |
| [IGFN1](http://www.ncbi.nlm.nih.gov/gene/?term=IGFN1) | 91156 | -2.3 | 3.3E-04 | - | - |
| [MXRA5](http://www.ncbi.nlm.nih.gov/gene/?term=MXRA5) | 25878 | -2.4 | 7.3E-05 | - | - |
| [SYT15](http://www.ncbi.nlm.nih.gov/gene/?term=SYT15) | 83849 | -2.5 | 5.9E-05 | - | - |
| [NR4A2](http://www.ncbi.nlm.nih.gov/gene/?term=NR4A2) | 4929 | -2.6 | 2.1E-05 | - | - |
| stanniocalcin 1 | 6781 | - | - | 3.2 | 6.9E-04 |
| matrix metallopeptidase 14 (membrane-inserted) | 4323 | - | - | 2.6 | 7.4E-04 |
| arachidonate 5-lipoxygenase-activating protein | 241 | - | - | 2.5 | 3.0E-04 |
| serpin peptidase inhibitor, clade E (nexin, plasminogen activator inhibitor type 1), member 2 | 5270 | - | - | 1.9 | 1.6E-03 |
| P antigen family, member 5 (prostate associated) | 90737 | - | - | 1.9 | 3.0E-02 |
| zinc finger protein 429 | 353088 | - | - | 1.9 | 2.5E-03 |
| thrombospondin 1 | 7057 | - | - | 1.8 | 3.8E-02 |
| membrane metallo-endopeptidase | 4311 | - | - | 1.8 | 1.2E-02 |
| podoplanin | 10630 | - | - | 1.8 | 2.4E-02 |
| serologically defined colon cancer antigen 8 | 10806 | - | - | 1.8 | 2.6E-02 |
| ZFP36 ring finger protein-like 1 | 677 | - | - | 1.8 | 3.2E-02 |
| family with sequence similarity 129, member B | 64855 | - | - | 1.8 | 7.7E-03 |
| ornithine decarboxylase 1 | 4953 | - | - | 1.8 | 2.9E-02 |
| Rho GTPase activating protein 18 | 93663 | - | - | 1.8 | 3.4E-02 |
| inhibin, beta A | 3624 | - | - | 1.8 | 2.6E-02 |
| nipsnap homolog 3B (C. elegans) | 55335 | - | - | -1.8 | 3.2E-02 |
| cyclin-dependent kinase inhibitor 1B (p27, Kip1) | 1027 | - | - | -1.8 | 1.2E-02 |
| plakophilin 2 | 5318 | - | - | -1.8 | 4.5E-02 |
| EGF-like repeats and discoidin I-like domains 3 | 10085 | - | - | -1.9 | 2.7E-03 |
| FAT atypical cadherin 4 | 79633 | - | - | -1.9 | 7.4E-03 |
| calsyntenin 2 | 64084 | - | - | -1.9 | 1.3E-02 |
| protein phosphatase methylesterase 1 | 51400 | - | - | -2.0 | 1.9E-03 |
| FH2 domain containing 1 | 85462 | - | - | -2.1 | 8.3E-03 |
| G protein-coupled receptor 162 | 27239 | - | - | -2.1 | 1.4E-03 |
| ring finger protein 144B | 255488 | - | - | -2.3 | 5.9E-04 |
| syndecan 2 | 6383 | - | - | -2.3 | 3.3E-04 |
| chemokine (C-C motif) ligand 2 | 6347 | - | - | -4.1 | 6.9E-04 |

^1^significant differences in gene expression were defined as fold change ≥ ±1.8 and FDR ≤ 0.05

**D**

**C**

**B**

**A**

**Supplementary Figure 1: Gating strategy for cell adhesion assay.** Given the substantial size difference between cancer cells and platelets a side scatter (SSC) v forward scatter (FCS) plot was used to differentiate between the two populations. **(A)** An unstained cancer cell sample was used to gate the cancer cell population on a SSC v FCS plot (P1). This P1 gated population was then selected and viewed on APC v PE plot (log scale). The unstained cancer cells were used to establish a gate for APC negative v positive cancer cells (P11 gate is APC positive cells). **(B)** A platelet sample incubated with an APC isotype control was then used to establish the platelet population on a SSC v FCS plot (P1). **(C)** A cancer cell sample incubated with an APC isotype control was used to refine the P11 gate (APC positive cancer cells). **(D)** Cells co-incubated with platelets and then CD42b-APC antibody, platelet marker, were then analyzed, with the % in the P11 gate equaling the % of cancer cells with platelets adhered.


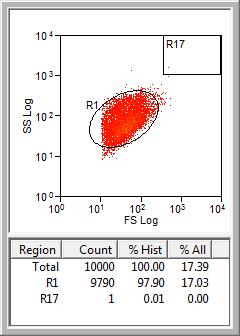

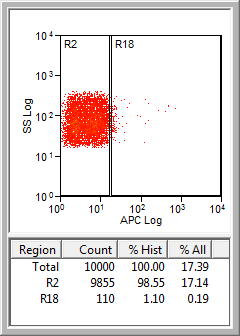

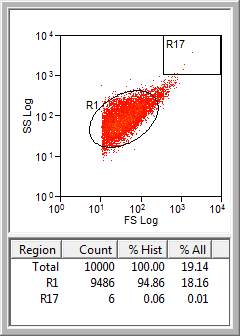

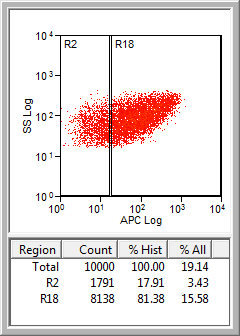

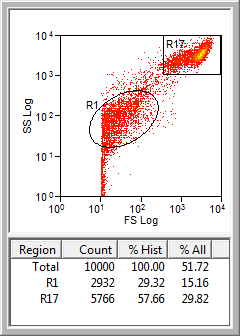

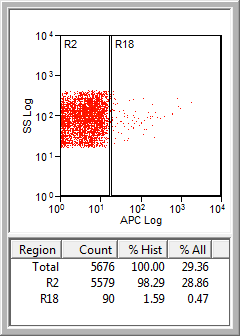

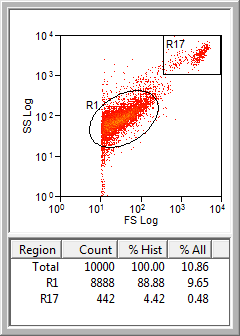

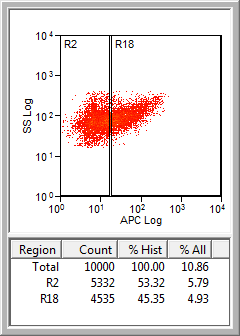


**C**

**B**

**A**

**D**

**Supplementary Figure 2: Gating strategy for cell activation assay.** Given the substantial size difference between cancer cells and platelets a side scatter (SS) v forward scatter (FS) plot was used to differentiate between the two populations. **(A)** A platelet sample co-incubated with an APC isotype control antibody was used to gate the platelet population on a SS v FS plot (R1). This R1 gated population was then selected and viewed on APC v SS plot (log scale). The APC isotype control sample was used to establish a gate for APC negative v positive platelets (R18 gate is APC positive platelets). **(B)** A platelet sample co-incubated with TRAP and then incubated with a P-selectin APC antibody was then used as a positive control for the assay **(C)** A cancer cell sample incubated with an APC isotype control antibody was used to identify the cancer cell population on the SS v FS plot. **(D)** Platelets co-incubated with cancer cells and then incubated with a P-selectin-APC antibody, platelet activation marker, were then analyzed with the % in the P18 gate equaling the % platelet activation.

**Supplementary Figure 3: No correlation was observed between platelet adhesion and activation assay results.** Platelet adhesion to cancer cells was quantified based on the fluorescence detection of CD42b positive platelets (n=4). Platelet activation by cancer cells was quantified based on the fluorescence detection of P-selectin (CD62P) positive platelets (n=3).


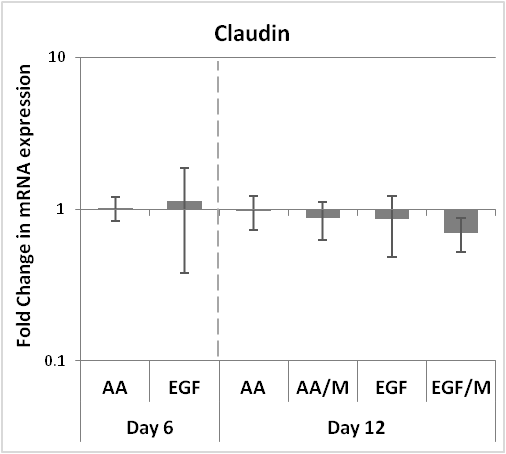

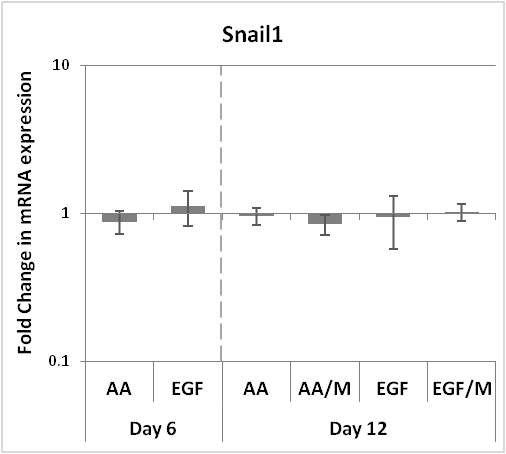


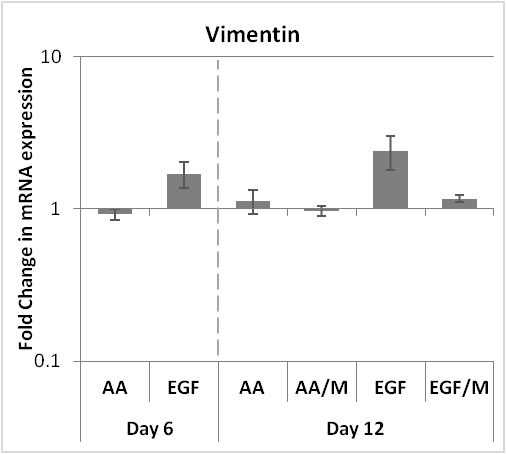


**Supplementary Figure 4. Effect of EGF treatment on expression of the epithelial and mesenchymal markers in SKOV3 cells.** SKOV3 cells were treated for 6 days with either 10 ng/ml EGF, AA or left untreated. On day 6 cells grown in EGF were continued in media with EGF (EGF) or without (EGF/M). Cells grown in AA were continued in media with AA (AA) or without (AA/M). Results are expressed as fold changes in mRNA expression of the mesenchymal associated genes, Snail1 and Vimentin, and the epithelial associated gene, Claudin, in the presence of EGF or AA (vehicle control) compared to untreated cells (n=3). Values were normalised to the endogenous control B2M. Data shown are mean values ± standard deviation. Significance was determined using Student’s *t* test, where *p≤ 0.05, ** p ≤ 0.01, *** p≤ 0.001.


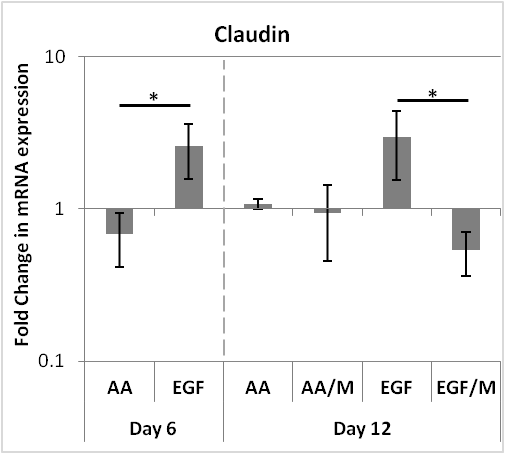

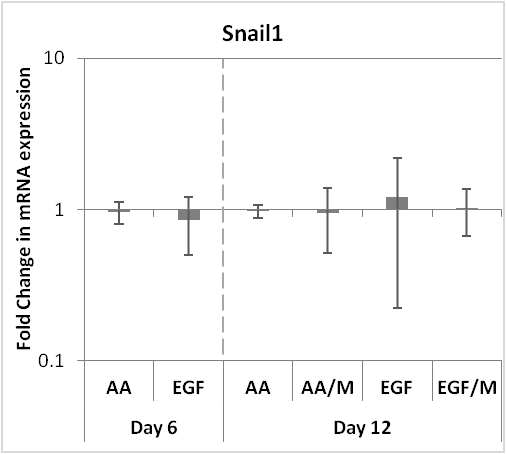


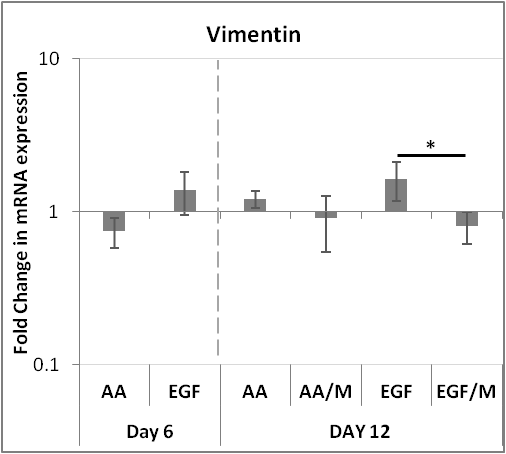


**Supplementary Figure 5. Effect of EGF treatment on expression of the epithelial and mesenchymal markers in 59M cells.** 59M cells were treated for 6 days with either 10 ng/ml EGF, AA or left untreated. On day 6 cells grown in EGF were continued in media with EGF (EGF) or without (EGF/M). Cells grown in AA were continued in media with AA (AA) or without (AA/M). Results are expressed as fold changes in mRNA expression of the mesenchymal associated genes Snail1 and Vimentin, and the epithelial associated gene, Claudin, in the presence of EGF or AA (vehicle control) compared to untreated cells (n=3). Values were normalised to the endogenous control GAPDH. Data shown are mean values ± standard deviation. Significance was determined using Student’s *t* test, where *p≤ 0.05, ** p ≤ 0.01, *** p≤ 0.001.

**
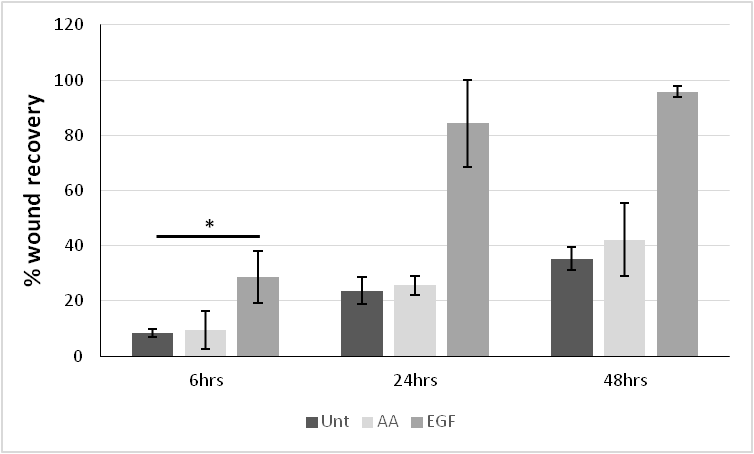
**

**B**

**A**


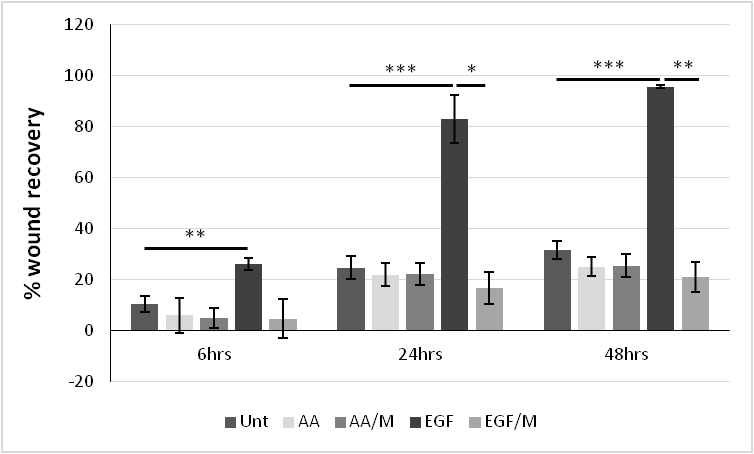


**Supplementary Figure 6: Scratch assay with SKOV3 cells.** Wound healing assay was performed on the SKOV3 cells on **(A)** day 6 and **(B)** day 12**.** Images were taken at 0, 6, 24 and 48 hour time points. Wound recovery was evaluated using ImageJ software, with the wound area at 0 hours for each condition and replicate defined as 0% recovery. Data shown are mean values ± SD (n=3). Significance was determined using Student’s *t* test, where *p≤ 0.05. ** p≤ 0.01, *** p≤ 0.001.


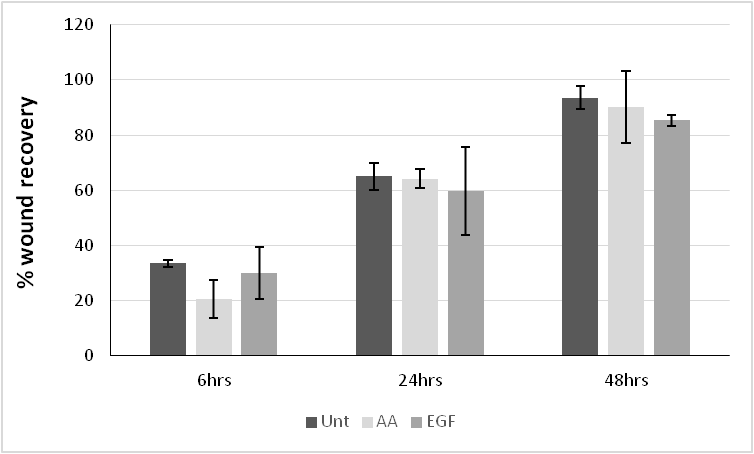


A

**Supplementary Figure 7. Scratch assay with 59M.** 59M cells were treated in 6 well plates for 4 days with either 10 ng/ml Epidermal growth factor (EGF), vehicle control (AA) or left untreated (NCM). Scratch assay was performed on the 59M cells after 4 days of treatment. Images were taken at 0, 6, 24 and 48 hour time points. Wound recovery was evaluated using ImageJ software, with the wound area at 0 hours for each condition and replicate defined as 0% recovery. Data shown are mean values ± standard deviation (n=3). Significance was determined using Student’s *t* test, where *p≤ 0.05. ** p ≤ 0.01, *** p≤ 0.001.


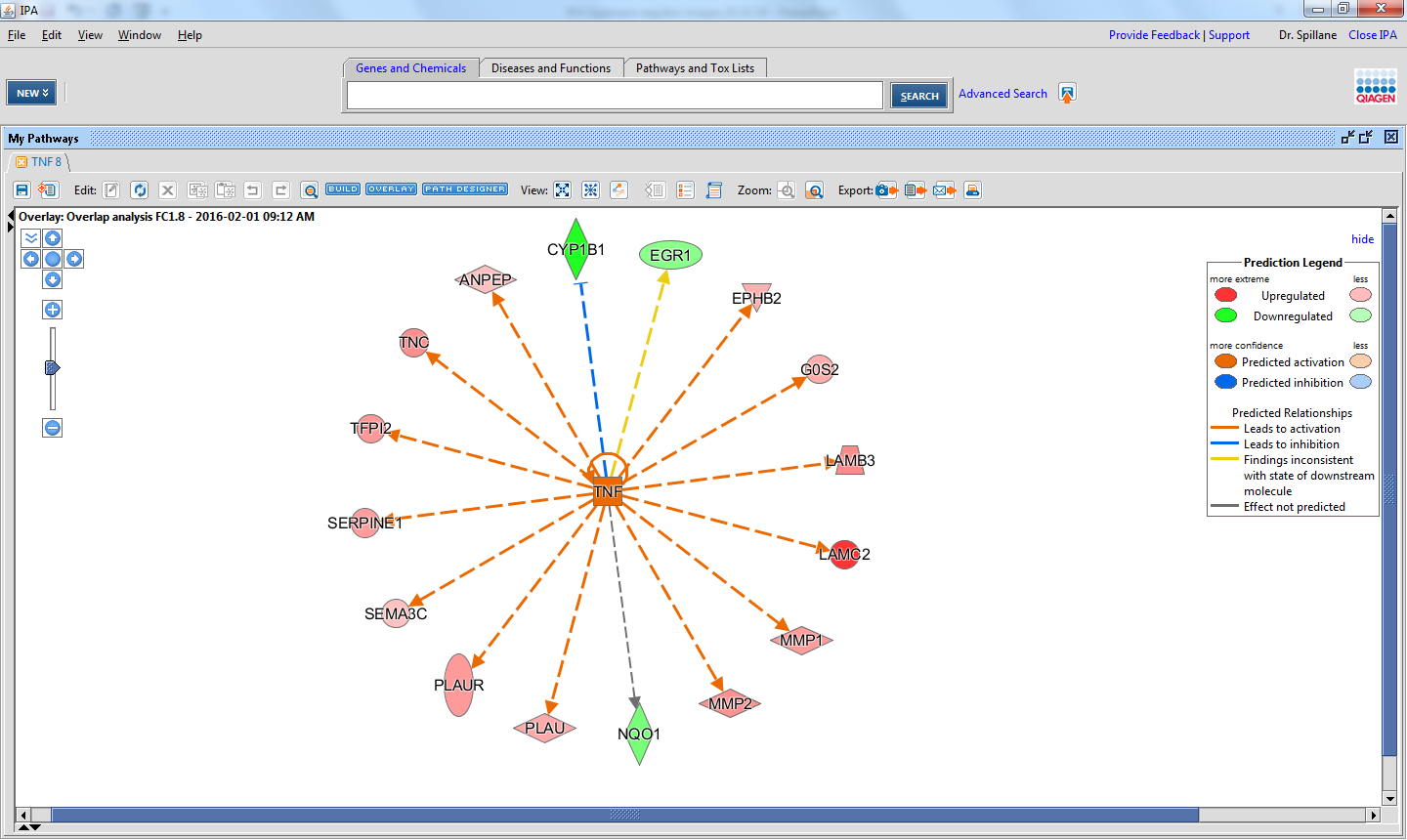


**Supplementary Figure 8.** Upstream regulators of the 34 gene signature. Examples of key regulators TGFb and TNF.
